# Supplementary material for: K128 ubiquitination constrains RAS activity by expanding its binding interface with GAP proteins
Source: EMBO J. 2024 Jun 10;43(14):2862–77. doi: 10.1038/s44318-024-00146-w (PMC11251195; doi:10.1038/s44318-024-00146-w)
Supplement: Supplementary file 1 — Appendix [file 44318_2024_146_MOESM1_ESM.pdf]

K128 ubiquitination constrains RAS activity by expanding the binding interface with GAP proteins

Appendix

Appendix Figure S1. Mono- and di-ubiquitination at K128 is the most prevalent post-translational modification of NRAS and KRAS. A-C

Appendix Figure S2. Construction of the ubiquitinated NRAS systems.

Appendix Figure S3. Chemical conjugation of ubiquitin to NRAS.

Appendix Figure S4. Modeling of 128-ubiquitinated KRAS with full-length NF1.

Appendix Figure S5. Construction of the ubiquitinated KRAS/NF1-GRD complex.

Appendix Figure S6. Ubiquitination of RAS at different sites

Appendix Figure S7. Validation of the CRISPR single-cell clones

Appendix Figure S8. The effect of K128 ubiquitination on MAPK signaling is GAP-dependent.

Appendix Figure S9. K128 ubiquitination alters RAS downstream signaling in SW1990 cells.

Appendix Table S1. Plasmids, antibodies, and oligos used in this study

A

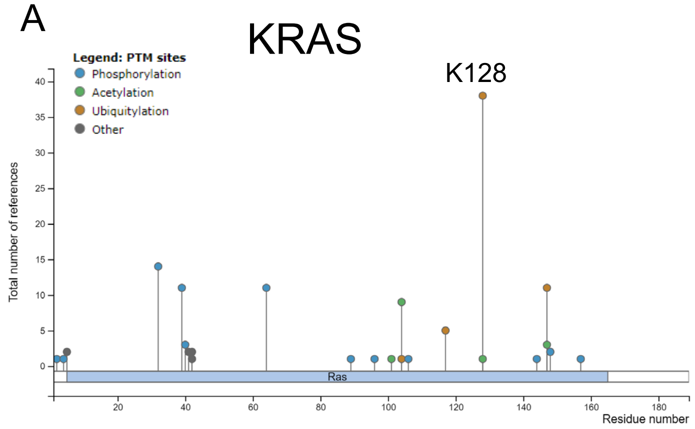

B

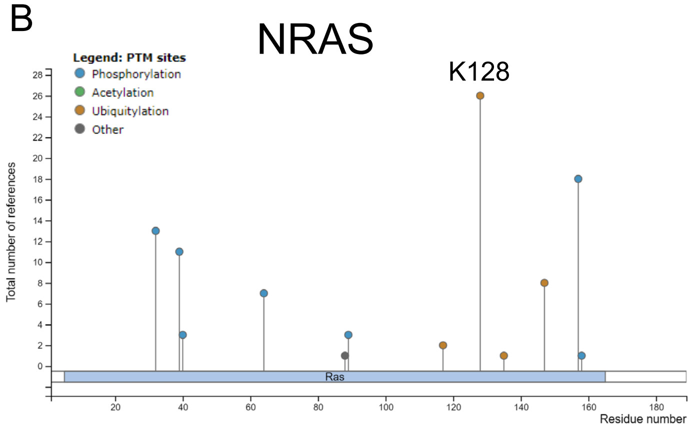

C

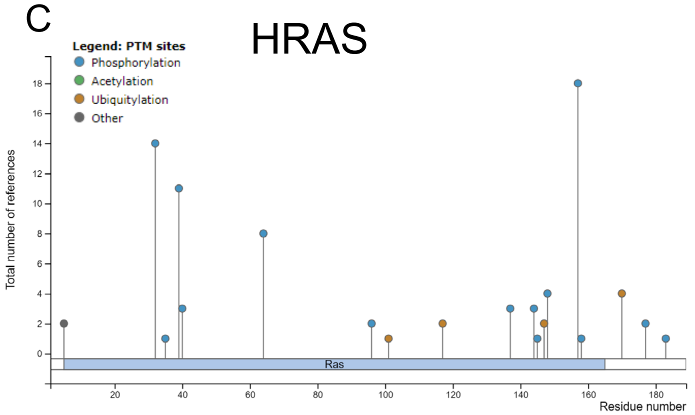

D

NRAS

Number of identified spectra:	K104	K117	K128	K135	K147
G-G-modified K	3	6	6	8	1
Non-modified K	27	41	9	18	18

KRAS

Number of identified spectra:	K104	K117	K128	R135	K147
G-G-modified K	3	0	11	-	7
Non-modified K	32	42	11	11	11

E

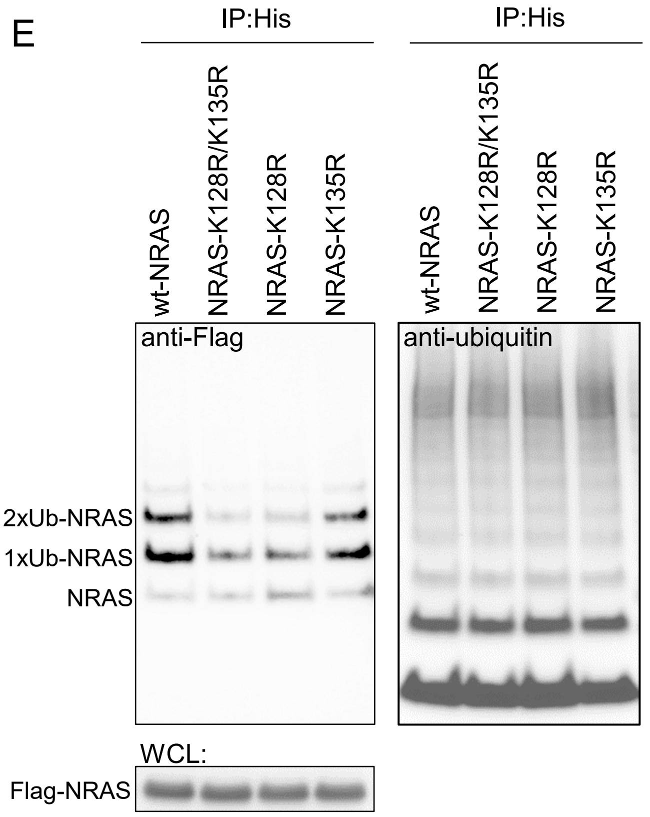

Appendix Figure S1. Mono- and di-ubiquitination at K128 is the most prevalent post-translational modification of NRAS and KRAS. A-C, Posttranslational modification profiles of KRAS **(A)**, NRAS **(B)**, and HRAS **(C)** proteins obtained from PhosphoSitePlus®. **D,** Identification of NRAS and KRAS ubiquitination sites by MS. **E,** Ubiquitinated wild-type and mutant NRAS proteins were purified by Co^{2+} metal affinity chromatography and detected by antibodies specific to Flag. Pull-down of His-tagged ubiquitinated proteins, His-Ub. Whole cell lysate, WCL.

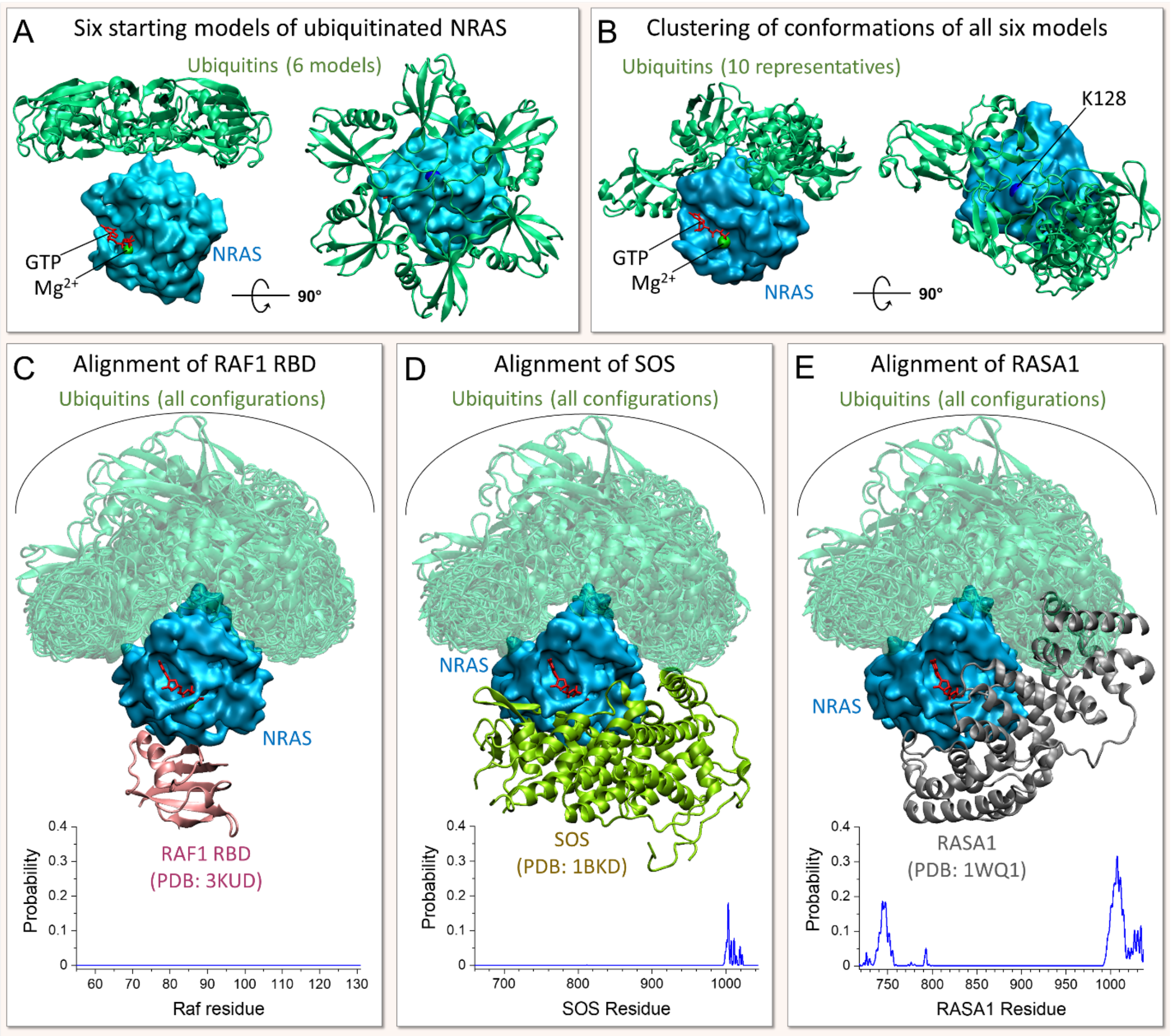

Appendix Figure S2. Construction of the ubiquitinated NRAS systems. A, Six different initial configurations of ubiquitinated NRAS for the all-atom MD simulations. NRAS is shown as a blue surface, and ubiquitins are shown as green cartoons. **B,** Superimpositions of the top ten representative conformations with respect to NRAS from the ensemble clusters for the simulated ubiquitinated NRAS system. **C,** Structural alignment of the RAF1 RBD (PDB: 3KUD) with the simulated ubiquitinated NRAS system and the probability of the RAF1 RBD contacting ubiquitin. The transparent cartoon shows all ubiquitin configurations for six models. **D,** The same for SOS (PDB: 1BKD), and **E,** the same for RASA1 (PDB: 1WQ1).

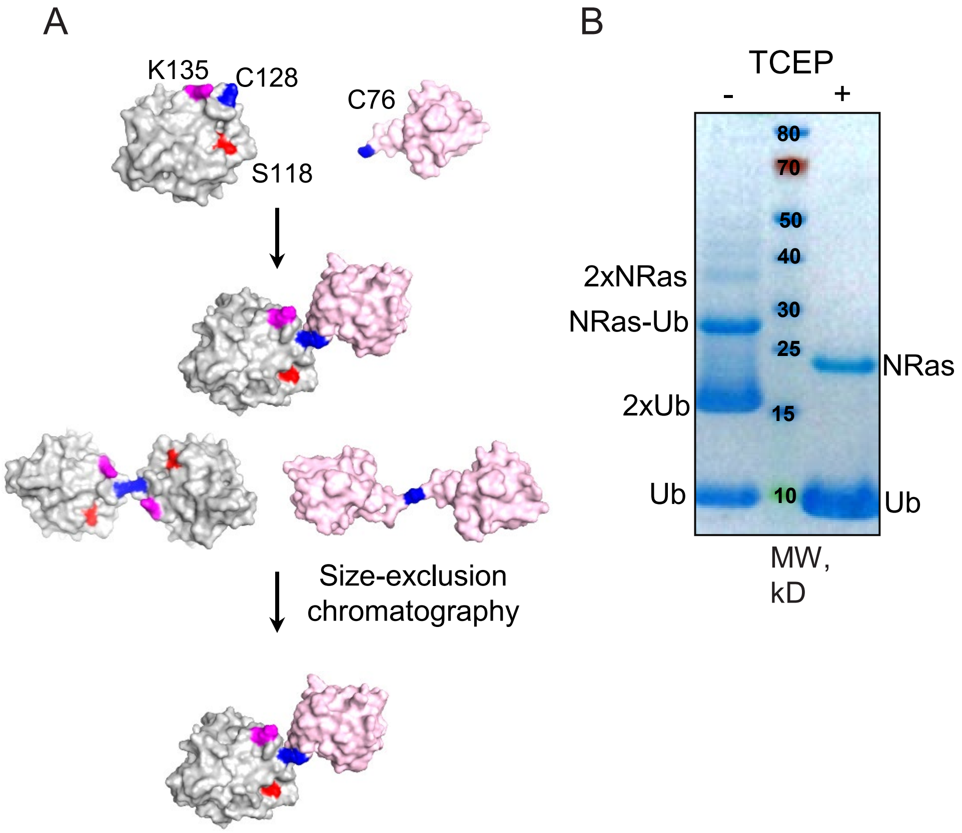

Appendix Figure S3. Chemical conjugation of ubiquitin to NRAS. A, Schematic outline of the chemical ubiquitination approach. Ubiquitin modification of NRAS-C118S (red)/K128C (blue) was performed by the addition of an excess of ubiquitin-G76C (blue). NRAS conjugated with ubiquitin was purified by size-exclusion chromatography. K135 is shown violet. **B,** Chemical ubiquitination of NRAS-C118S/ K128C mutant. Proteins were separated by SDS-PAGE under non–reducing conditions or in the presence of a reducing agent, tris(2-carboxyethyl)phosphine (TCEP) and stained by Coomassie blue.

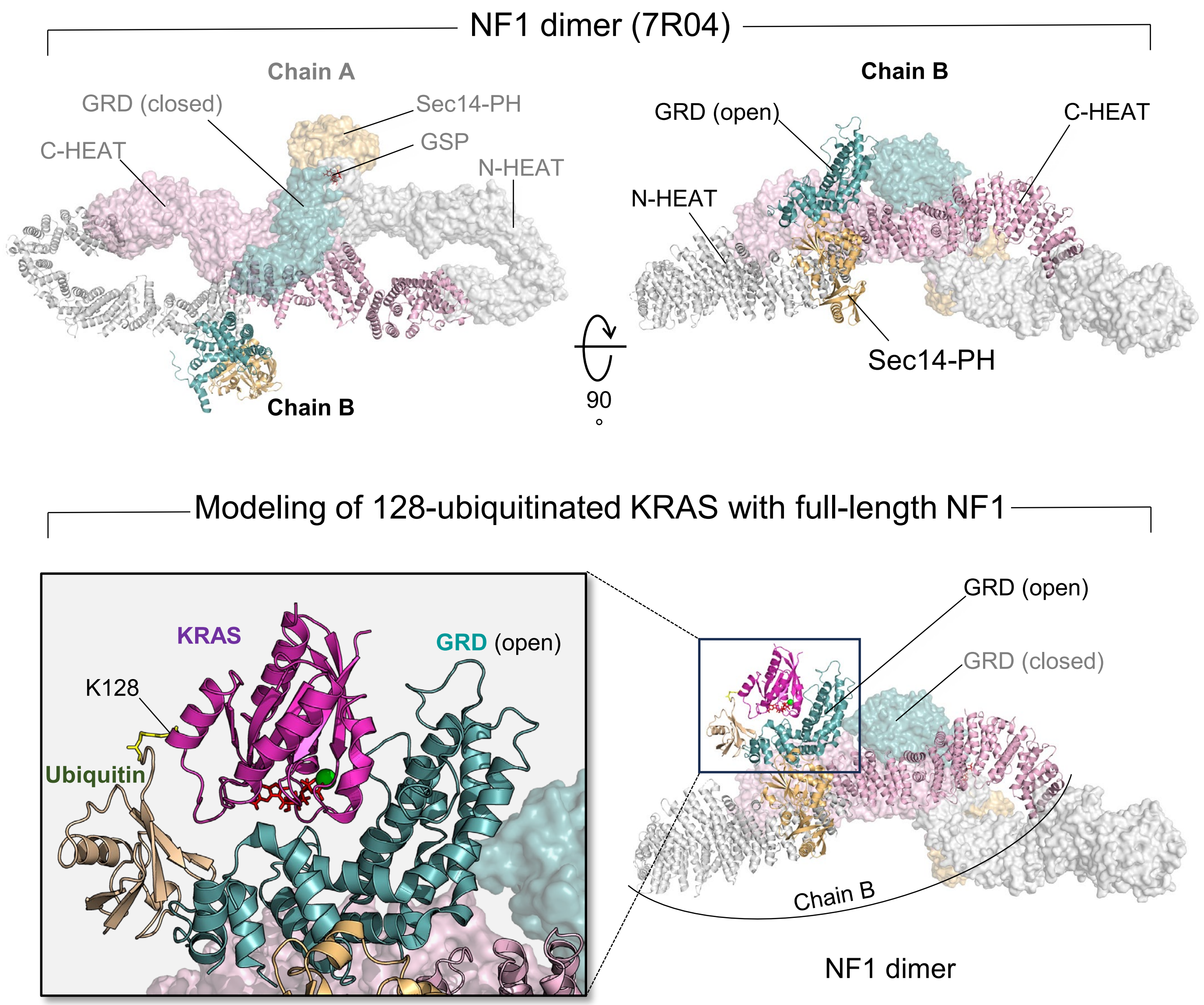

Appendix Figure S4. Modeling of 128-ubiquitinated KRAS with full-length NF1. Cryoelectron microscopy (cryo-EM) structures of the full-length NF1 homodimer (PDB:7R04, top panel). The NF1 dimer shows both an occluded (chain A, transparent surface) and an open (chain B, cartoon) conformation. In the occluded conformation of NF1, GRD has a closed RAS binding site, whereas in the open conformation, GRD is exposed for RAS binding. Modeling of simulated 128-ubiquitinated KRAS with the cryo-EM structure of NF1 (bottom panel). The coordinates of the simulated systems were aligned to the NF1 GRD (chain B). No steric crash between the ubiquitin and NF1 was observed.

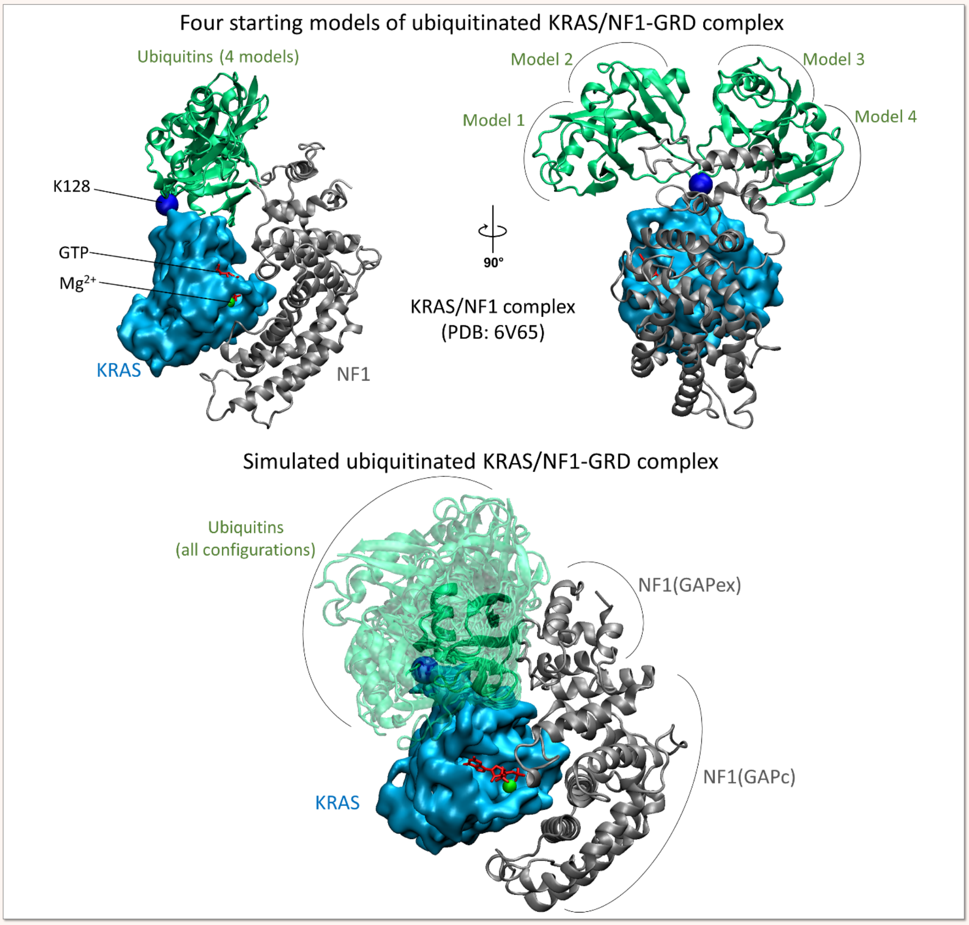

Appendix Figure S5. Construction of the ubiquitinated KRAS/NF1-GRD complex. Four different initial configurations of the ubiquitinated KRAS/NF1-GRD complex. The crystal structure of the non-ubiquitinated KRAS/NF1-GRD complex (PDB: 6V65) was used to construct the ubiquitinated systems. The initial ubiquitin positions were taken from the ubiquitinated NRAS systems. KRAS is shown as a blue surface. Ubiquitin and NF1-GRD are shown as green and gray cartoons, respectively. Superimpositions of simulated complex conformations for the ubiquitinated KRAS/NF1-GRD systems. The transparent cartoon shows all ubiquitin configurations, and the opaque ubiquitin indicates the best representative conformation from the ensemble clusters.

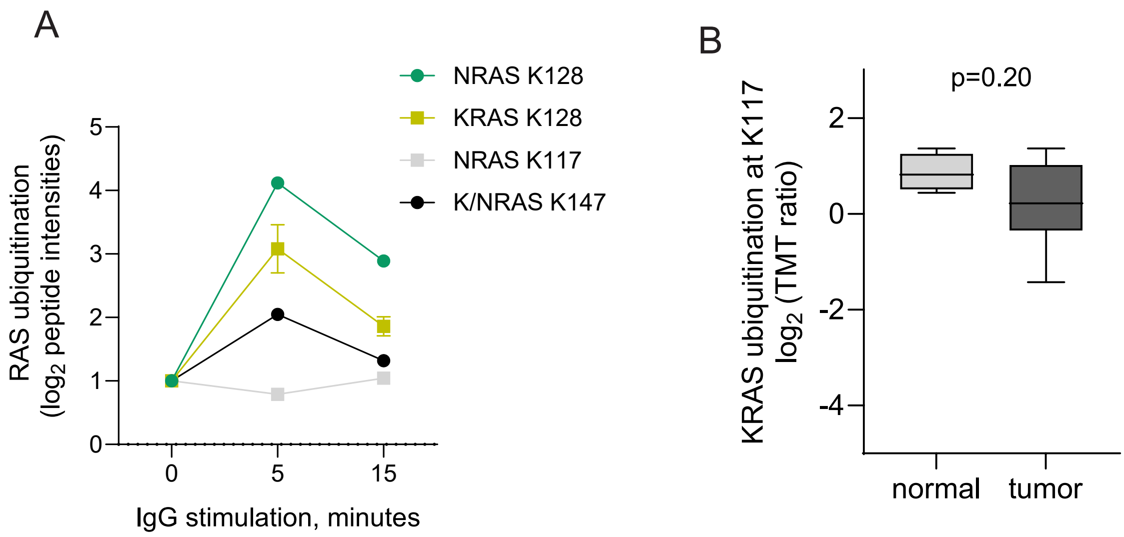

Appendix Figure S6. Ubiquitination of RAS at different sites. A, A20 cells were isotopically labeled using the SILAC approach and stimulated with α-IgG F(ab′)2 for 5 and 15 minutes. Ubiquitination levels of NRAS or KRAS at different sites detected by the MS-based ubiquitinome analysis. The data was obtained from Satpathy et al., 2015. **B,** K117 ubiquitination of KRAS detected by CPTAC ubiquitinome analysis in tumor (n = 85) and normal (n = 58) tissue of LUSC patients. Data are represented as box plots with the median as center, the interquartile range (IQR) indicated with a rectangular box and the whiskers are defined by the 1st and 3rd quartile ± 1.5x IQR. Data are shown as mean ± s.e.m. P - value is determined by a two-sided t-test.

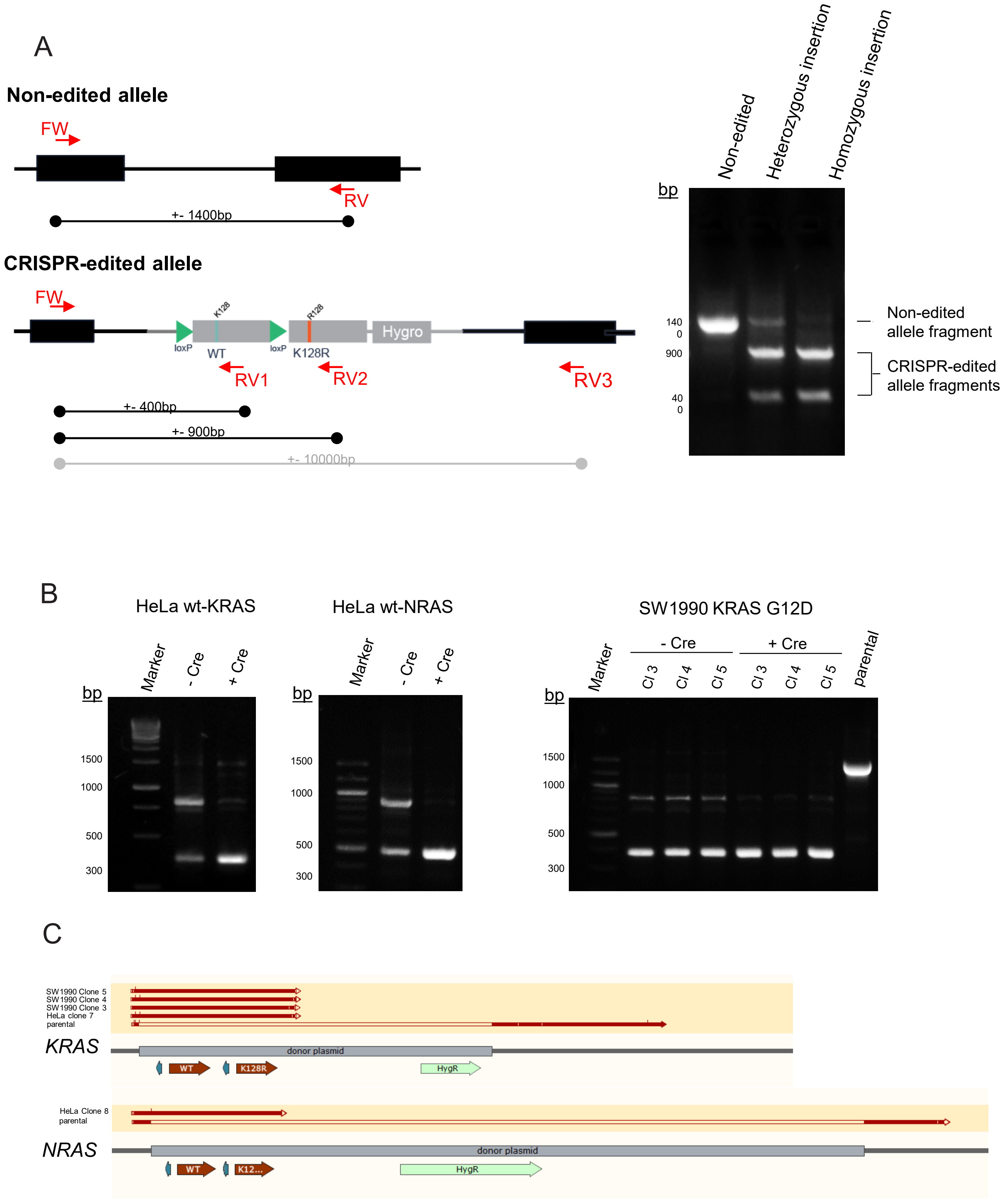

Appendix Figure S7. Validation of the CRISPR single-cell clones. A, Schematic illustration of the primer design and PCR strategy to confirm the integration of the cassette and to determine the zygosity of the insertion. **B,** Agarose gel electrophoresis of the PCR products for the different single-cell clones. **C,** Alignment of the Sanger sequencing of the single-cell clones with the CRISPR-edited allele sequence. bp, base pair.

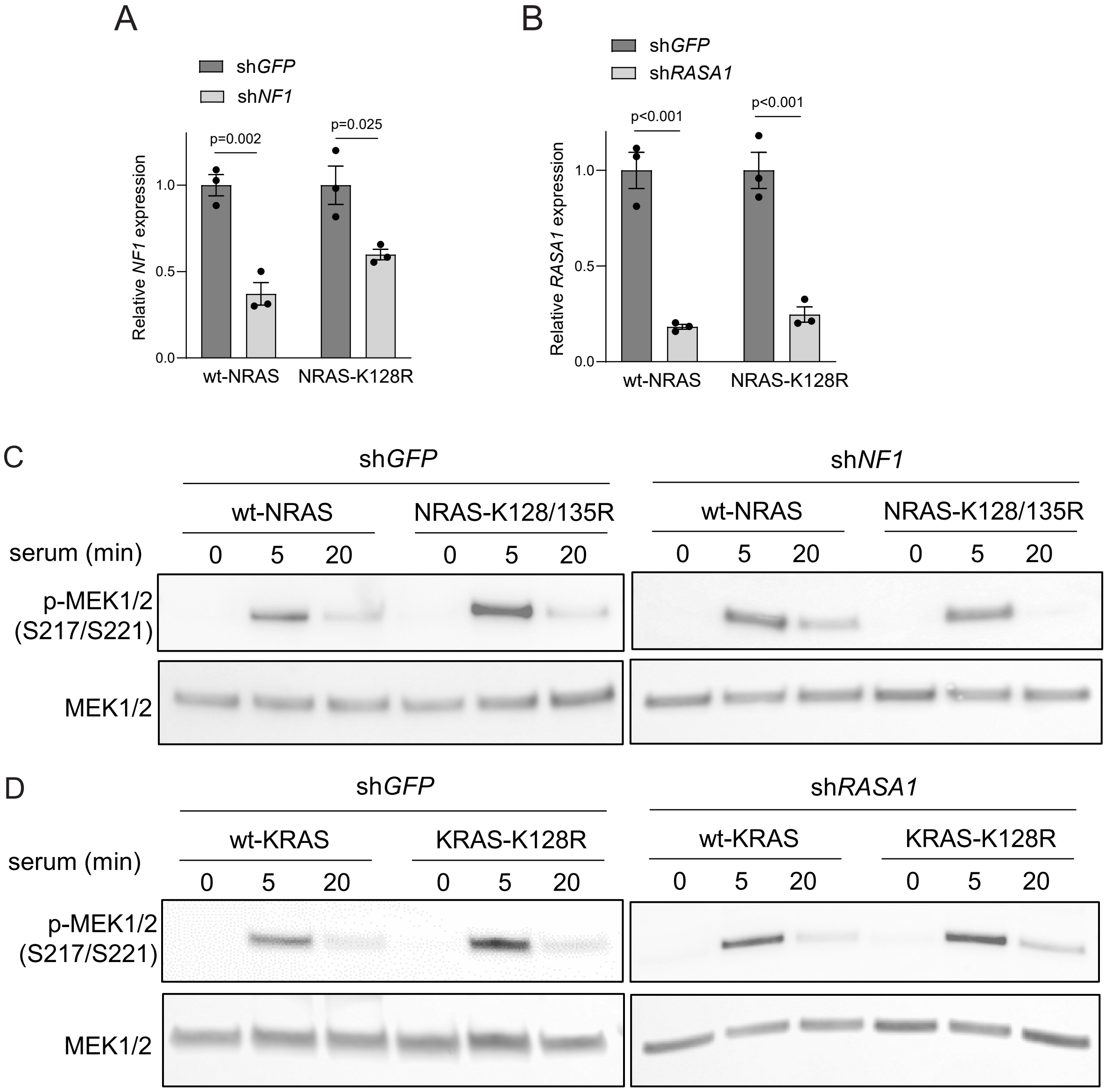

Appendix Figure S8. The effect of K128 ubiquitination on MAPK signaling is GAP-dependent. A,B, RT-qPCR analysis of *RASA1* and *NF1* in the indicated HeLa clones expressing either sh*GFP* or shRNA targeting *RASA1* or *NF1*. Data are present as mean ± SEM. n = 3 independent experiments. The P-value was calculated by a two-sided t-test. **C,D,** Immunoblot analysis of pMEK and MEK expression in the indicated HeLa clones expressing sh*GFP* or sh*NF1.* The HeLa cells were serum-starved overnight and stimulated with 10% serum.

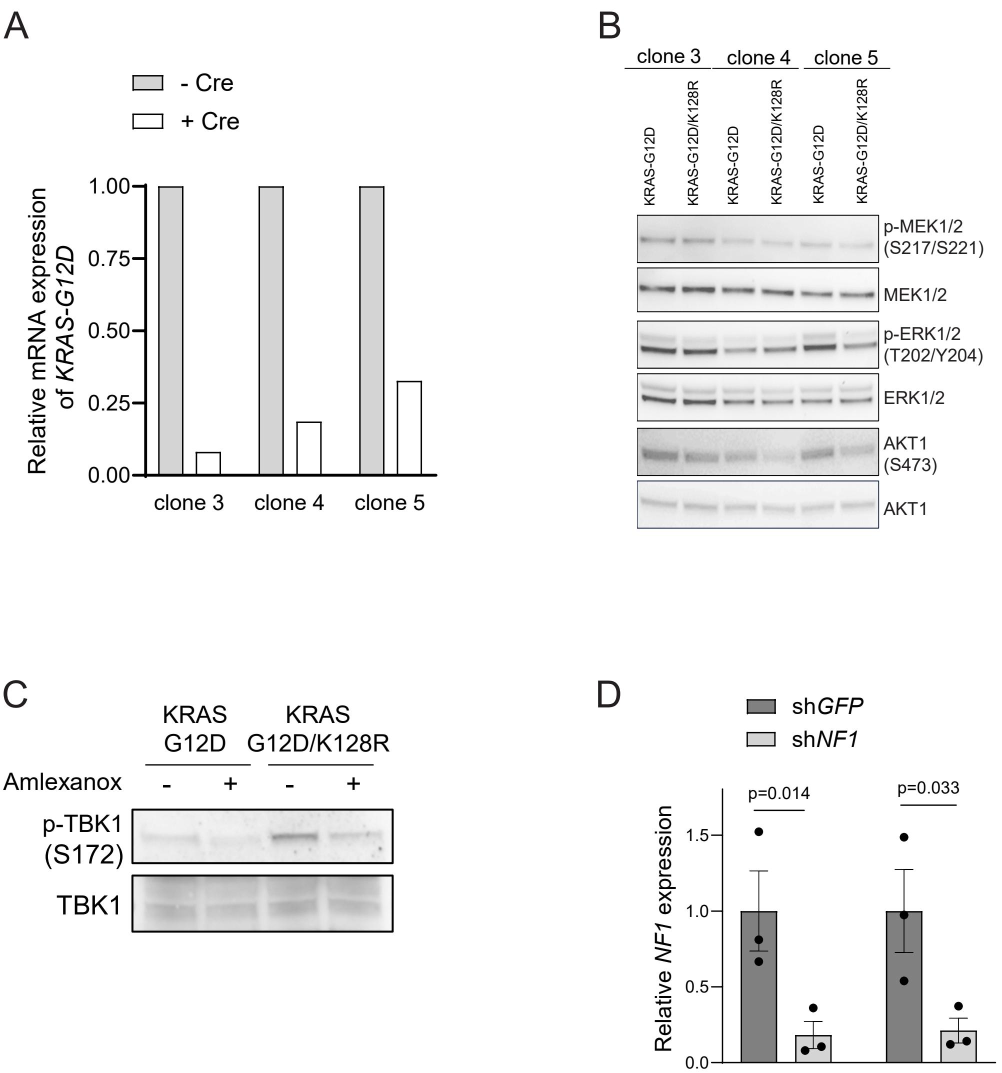

Appendix Figure S9. K128 ubiquitination alters RAS downstream signaling in SW1990 cells. A, Expression of *KRAS-G12D* in SW1990 single-cell clones harboring the *RAS* knock-in cassette before and after overexpression of Cre recombinase **B,** Immunoblot analysis of the indicated proteins in SW1990 single-cell clones expressing either KRAS-G12D or KRAS-G12D/K128R. **C,** Immunoblot analysis of phosphorylated and total TBK1 in SW1990 single-cell clones treated with DMSO or Amlexanox (25 μM, 24h). **D,** RT-qPCR analysis of *NF1* in the indicated SW1990 clones expressing either sh*GFP* or sh*NF1*. Data are present as mean ± SEM (n = 3). P - value was calculated by a two-sided t test.

Appendix Table S1. Plasmids, antibodies and oligos used in this study

Plasmids

plasmid name	Source
pMT107–6xHis–ubiquitin	a gift from Dr. Bohmann (University of Rochester, USA)
pLA-CMV-Flag-KRAS	created in the lab
pLA-CMV-Flag-KRAS K128R	
pLA-CMV-Flag-NRAS	created in the lab
pLA-CMV-Flag-NRAS K128R/K135R	
MSCV-CMV-DsRed-IRES-EGFP-DEST	a gift from Dr. Stephen Elledge (Addgene plasmid #41941)
MSCV-CMV-DsRed-IRES-EGFP-KRAS WT	created in the lab
MSCV-CMV-DsRed-IRES-EGFP-KRAS K128R	created in the lab
MSCV-CMV-DsRed-IRES-EGFP-NRAS WT	created in the lab
MSCV-CMV-DsRed-IRES-EGFP-NRAS K128R/K135R	created in the lab
Champion pET302/NT-His-DEST	Invitrogen (K6300-01)
Champion pET302/NT-His-ubiquitin G76C	created in the lab
Champion pET302/NT-His-TEV-Flag NRAS C118S/K128C	created in the lab
pF3A_WG_His-GST	Promega (L5671)
pF3A_WG_His-GST-RASA1	created in the lab
pF3A_WG_His-GST-RASA1 E1015A/R1016A	created in the lab
pGEX-4T2	GE Healthcare Life Sciences
pGEX-4T2-GST-NF1(GRD)	created in the lab
pGEX-4T2-GST-NF1(GRD) L1501A/D1506A/R1513A	created in the lab
pN-CAT-Target	generated in Prag lab
pN-CAT-RASA1(GAP)	created in the lab
pN-CAT-RASA1(GAP) E1015A/R1016A	created in the lab
pN-CAT-NF1(GRD)	created in the lab
pN-CAT-NF1(GRD) L1501A/D1506A/R1513A	created in the lab
pC-CAT-Ubiquitin	generated in Prag lab
pX330-CAS9	a gift from Feng Zhang (Addgene plasmid #42230)
pCRISPR KRAS K128R	Vectorbuilder
pCRISPR NRAS K128/135R	Vectorbuilder

shRNA constructs

plasmid name	Resource	TRC number	shRNA target sequence
pLKO.1-puro sh*RASA1*	Sigma Aldrich	TRCN0000005999	CCCTACATGGAAGGTGTCAAT
pLKO.1-puro sh*NF1*	Sigma Aldrich	TRCN0000039714	GCCAACCTTAACCTTTCTAAT

Antibodies

Antigen	Host species	Clonality	Clone number	Company	Reference
anti-Flag	Mouse	Monoclonal	M2	Sigma Aldrich	F3165
anti-RAS	Mouse	Monoclonal	RAS10	Millipore	05-516
anti-vinculin	Mouse	Monoclonal	hVIN-1	Sigma Aldrich	V9131
anti-Erk1/2	Mouse	Monoclonal	3A7	Cell Signaling Technologies	#9107
anti-Phospho-Erk1/2 (Thr202/Tyr204)	Rabbit	Polyclonal		Cell Signaling Technologies	#9101
anti-HA	Rat	Monoclonal	3F10	Roche	11867423001
Anti-His	Rabbit	Polyclonal		Santa Cruz Biotechnology	sc-803
anti-GST	Rabbit	Monoclonal	91G1	Cell Signaling Technologies	#2625
anti-ubiquitin	Rabbit	Polyclonal		Abcam	ab7780
anti-phospho-MEK1/2 (Ser217/221)	Rabbit	Polyclonal		Cell Signaling Technologies	#9121
anti-MEK1/2	Mouse	Monoclonal	L38C12	Cell Signaling Technologies	#4694
anti-RALB	Rabbit	Monoclonal	EPR6471	Abcam	ab129077
anti-SEC5	Rabbit	Polyclonal		Sigma Aldrich	HPA032093
anti-TBK1	Mouse	Monoclonal	E9H5S	Cell Signaling Technologies	#51872
anti-Phospho-TBK1	Rabbit	Polyclonal		Proteintech	82383-1-RR
anti-AKT1	Mouse	Monoclonal	40D4	Cell Signaling Technologies	#2920
anti-phospho-AKT (Ser473)	Rabbit	Monoclonal	D9E	Cell Signaling Technologies	#4060
Phospho-TBK1/NAK (Ser172)	Rabbit	Monoclonal	D52C2	Cell Signaling Technologies	#5483

qPCR primers

Gene Name	Forward primer	Reverse primer
KRAS wt allele	GAAAAGATGAGCAAAGATGG	TTATACGAAGTTATGGTGGC
NRAS wt allele	TCAGGGTTGTATGGGATTG	CATTATACGAAGTTATTGTC
RASA1	TTGCATGAGATTTGCGTGGC	TTCAATACGTGCTGCTGTGC
NF1	GCCTTGAGGAAAACCAGCGGAA	TCCTACTGCACCGATGCTGTTC
TBP	CGGCTGTTTAACTTCGCTTC	CACACGCCAAGAAACAGTG
HPRT1	TGACACTGGCAAAACAATGCA	GGTCCTTTTCACCAGCAAGCT
HMBS	GAAACTCTGCTTCGCTGCATT	TGCCCATCTTTCATCACTGTATG
CRE	CGAACGCACTGATTTCGACC	TGATCCTGGCAATTTCGGCT

gRNA sequences

Gene Name	gRNA sequence
KRAS	GCTCCAGACTGCATCGGTAG
NRAS	TCCTGATACATGACCTAGTG
Frame selector	GCCAGTACCCAAAAAGCGGG

Sequencing Primers

Gene Name	forward sequence	reverse sequence
KRAS	GAGTCTTTGCTAATGCCATGC	CCGAACACCATTAGCTGGGT
NRAS	TGAGCACCACCCATCTTGAAA	CTTATCATGTCTGCTCGAAGCG
