# Supplementary figures and images for: K128 ubiquitination constrains RAS activity by expanding its binding interface with GAP proteins
Source: EMBO J. 2024 Jun 10;43(14):2862–77. doi: 10.1038/s44318-024-00146-w (PMC11251195; doi:10.1038/s44318-024-00146-w)

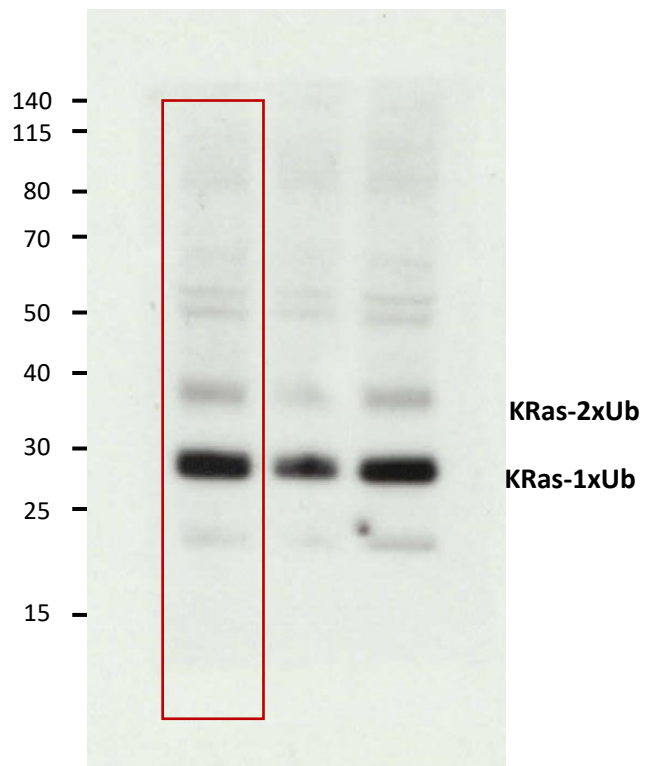

Supplement: Supplementary file 3 — Source data Fig. 1 [file 44318_2024_146_MOESM3_ESM.zip › Fig 1A-KRAS-TAP.pdf]

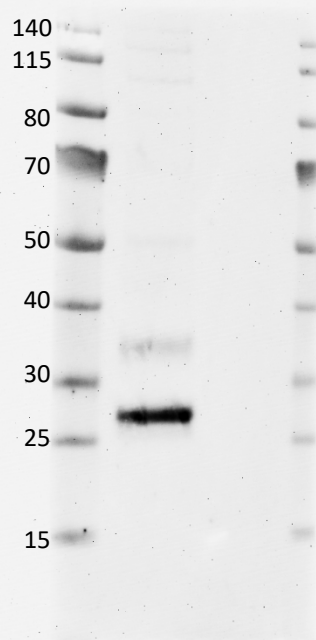

Supplement: Supplementary file 3 — Source data Fig. 1 [file 44318_2024_146_MOESM3_ESM.zip › Fig 1A-NRAS-TAP.pdf]

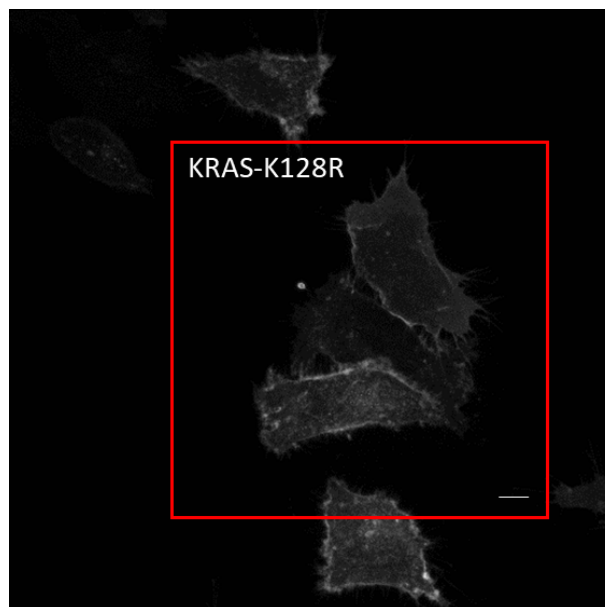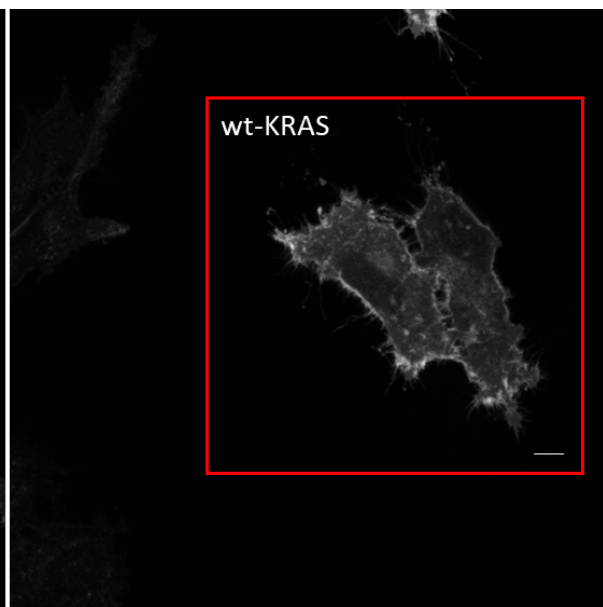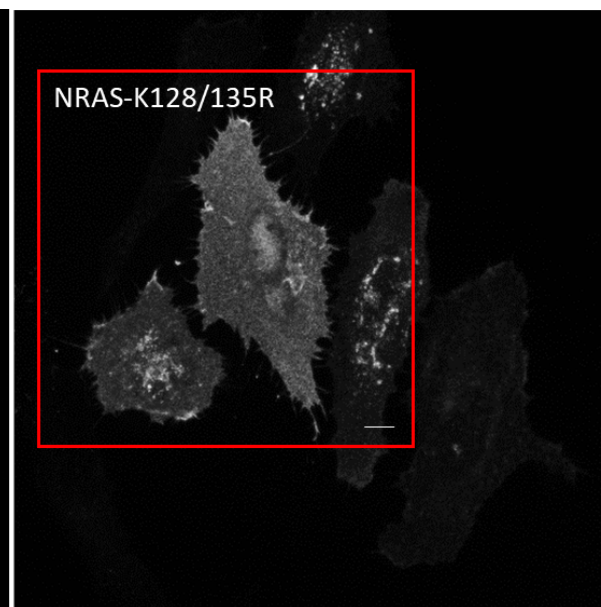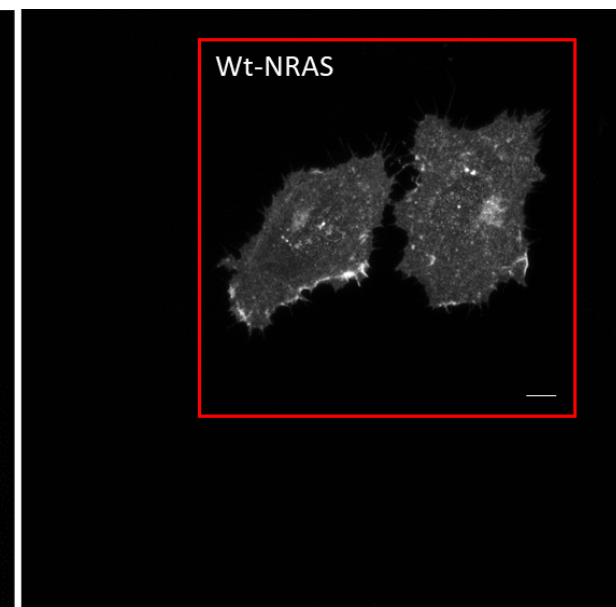

Supplement: Supplementary file 3 — Source data Fig. 1 [file 44318_2024_146_MOESM3_ESM.zip › Fig 1D-IF.pdf]

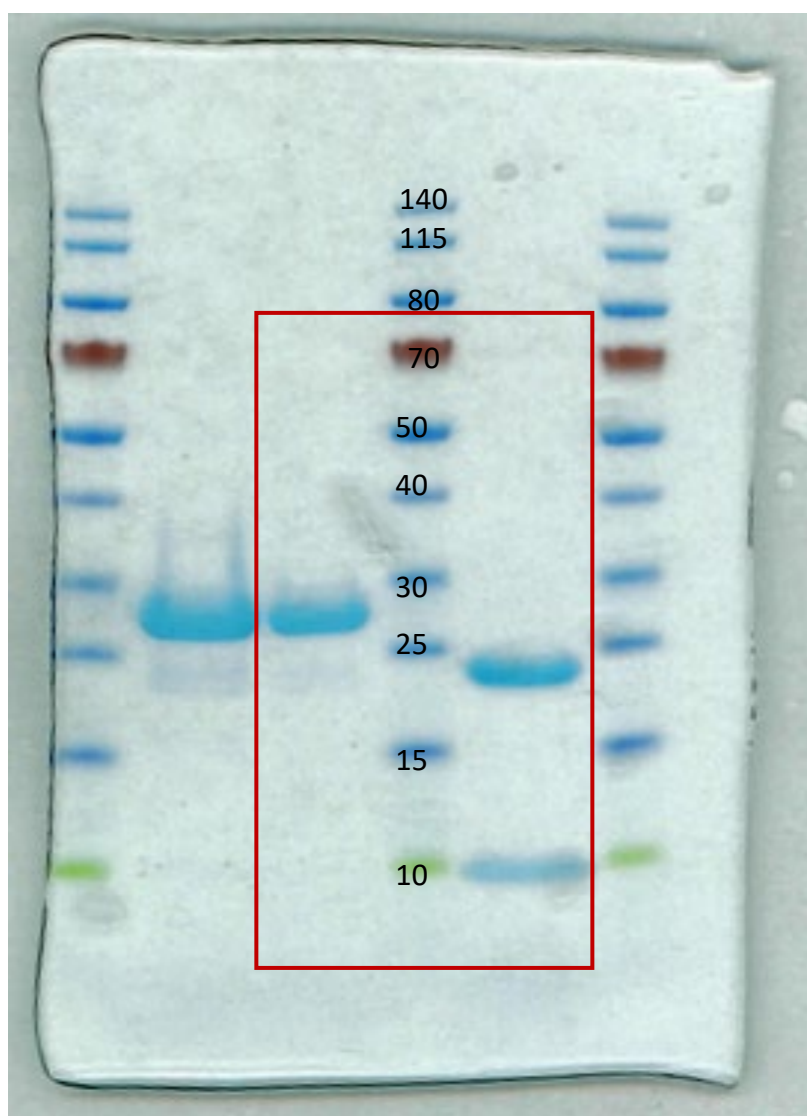

Supplement: Supplementary file 4 — Source data Fig. 2 [file 44318_2024_146_MOESM4_ESM.zip › Fig 2C- NRAS-Ub.pdf]

IP:Flag

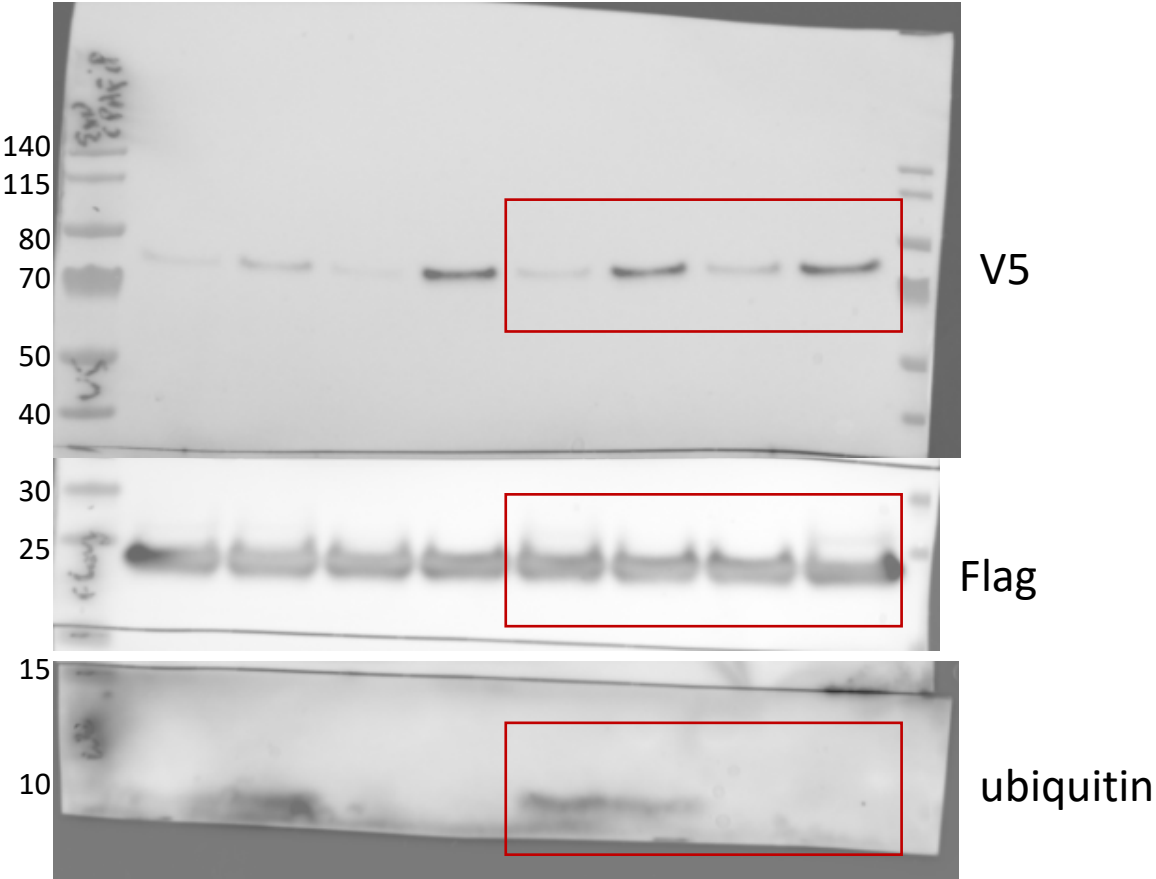

WCL:

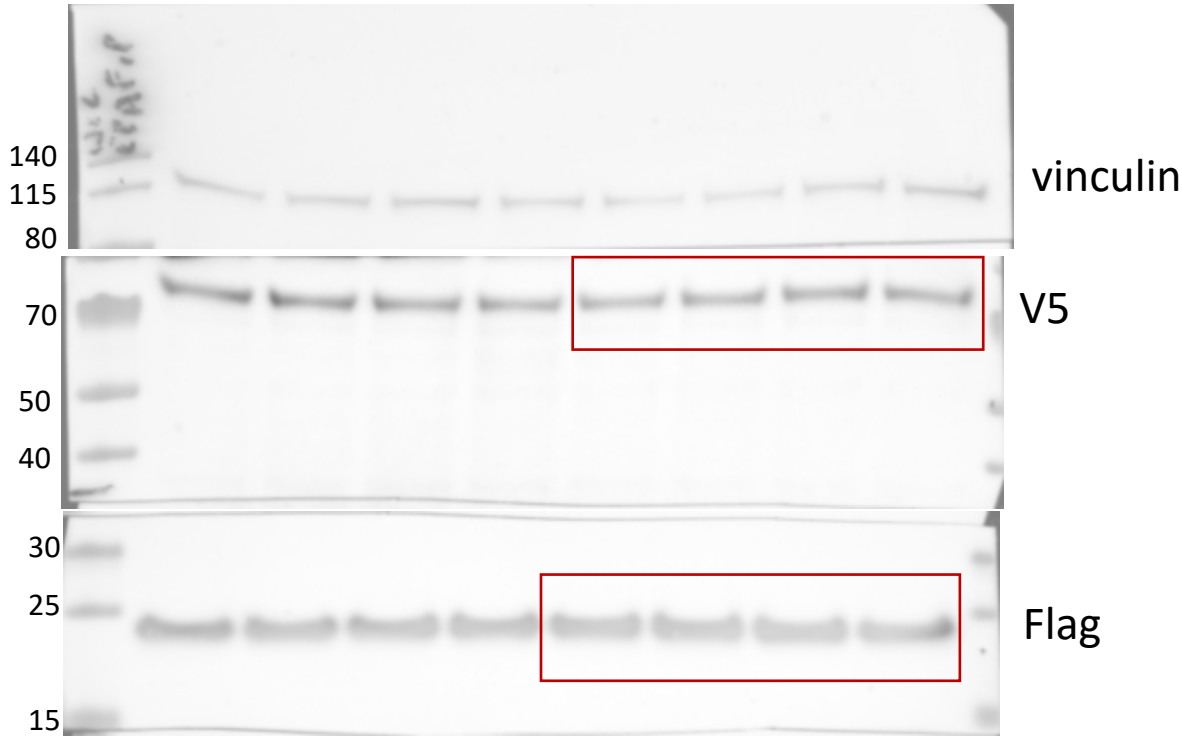

Supplement: Supplementary file 4 — Source data Fig. 2 [file 44318_2024_146_MOESM4_ESM.zip › Fig 2D-IP-WCL.pdf]

IP: Flag

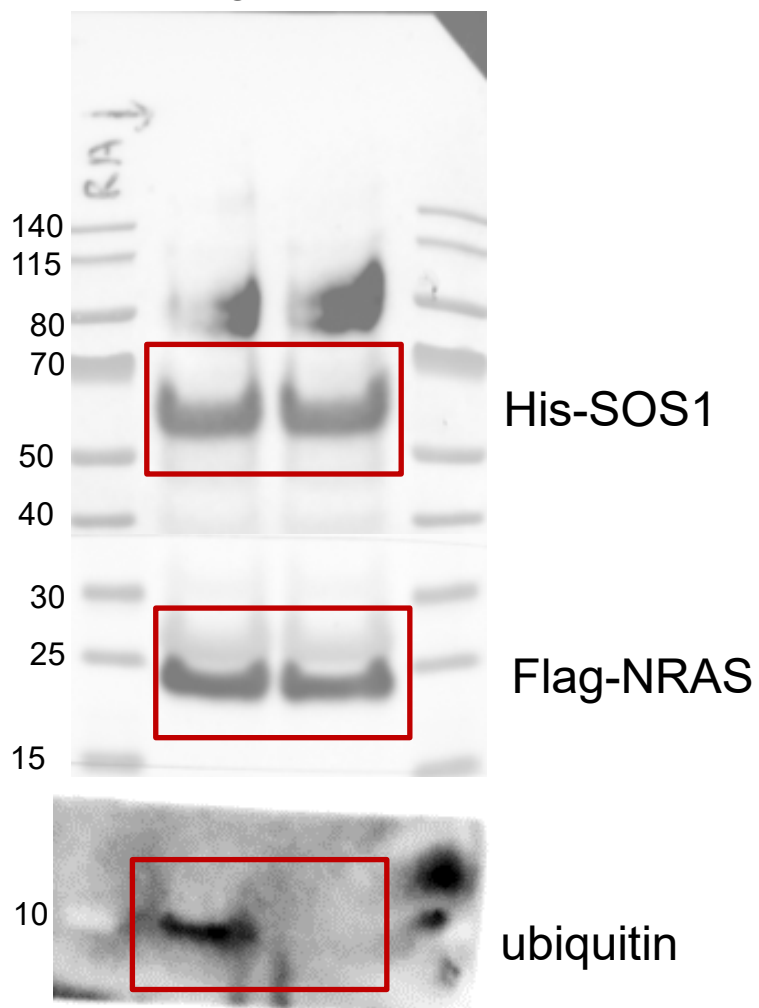

Supplement: Supplementary file 4 — Source data Fig. 2 [file 44318_2024_146_MOESM4_ESM.zip › Fig 2E -SOS1-IP.pdf]

WCL:

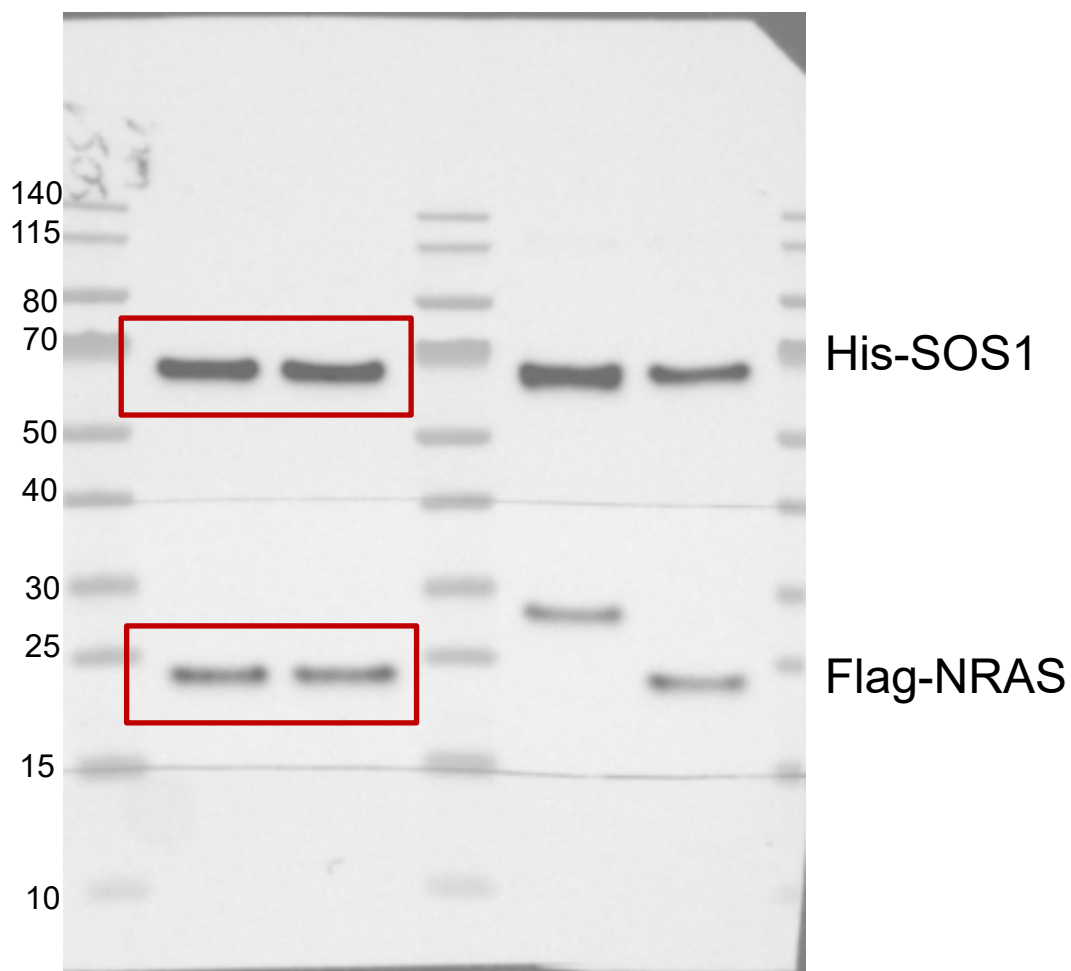

Supplement: Supplementary file 4 — Source data Fig. 2 [file 44318_2024_146_MOESM4_ESM.zip › Fig 2E- WCL.pdf]

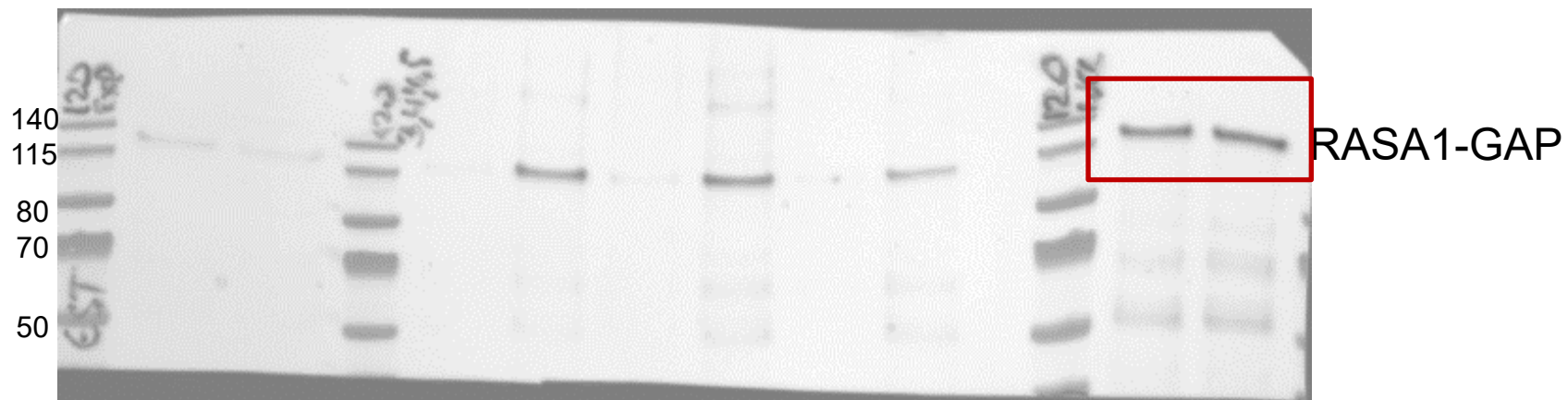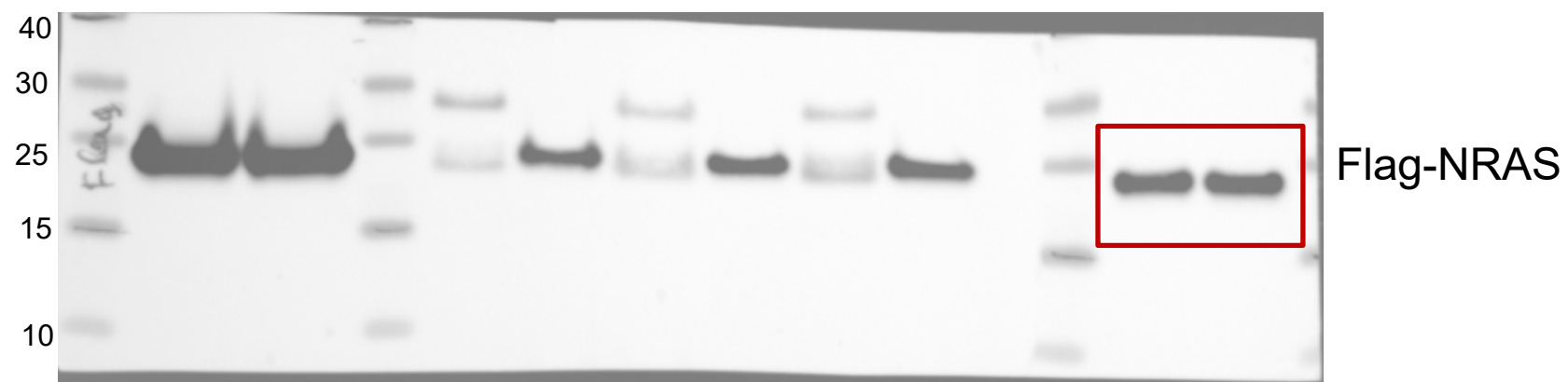

Supplement: Supplementary file 4 — Source data Fig. 2 [file 44318_2024_146_MOESM4_ESM.zip › Fig 2F -RASA1-WCL.pdf]

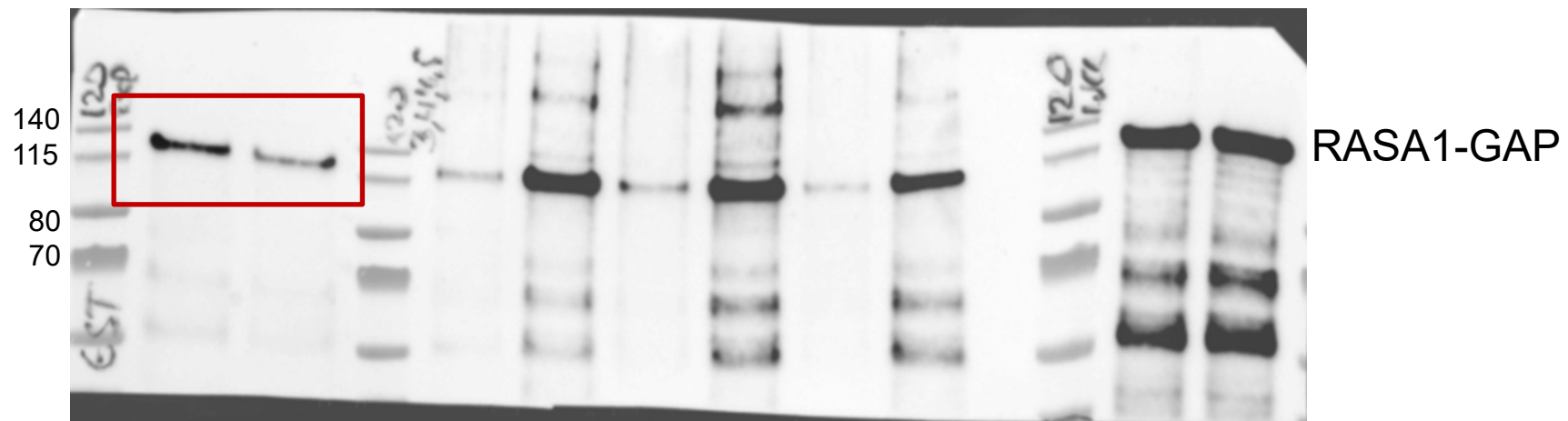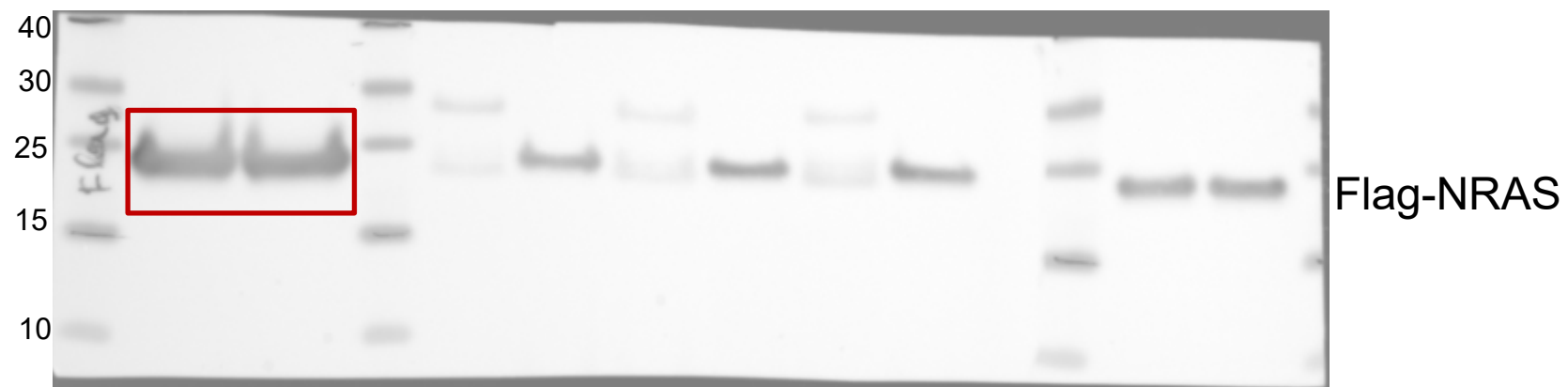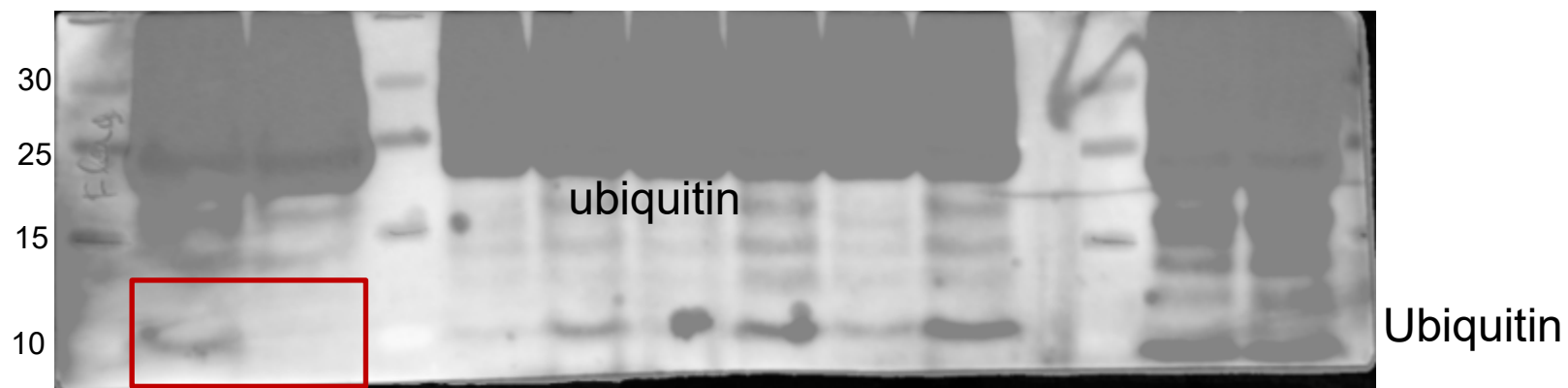

Supplement: Supplementary file 4 — Source data Fig. 2 [file 44318_2024_146_MOESM4_ESM.zip › Fig 2F-RASA1-IP.pdf]

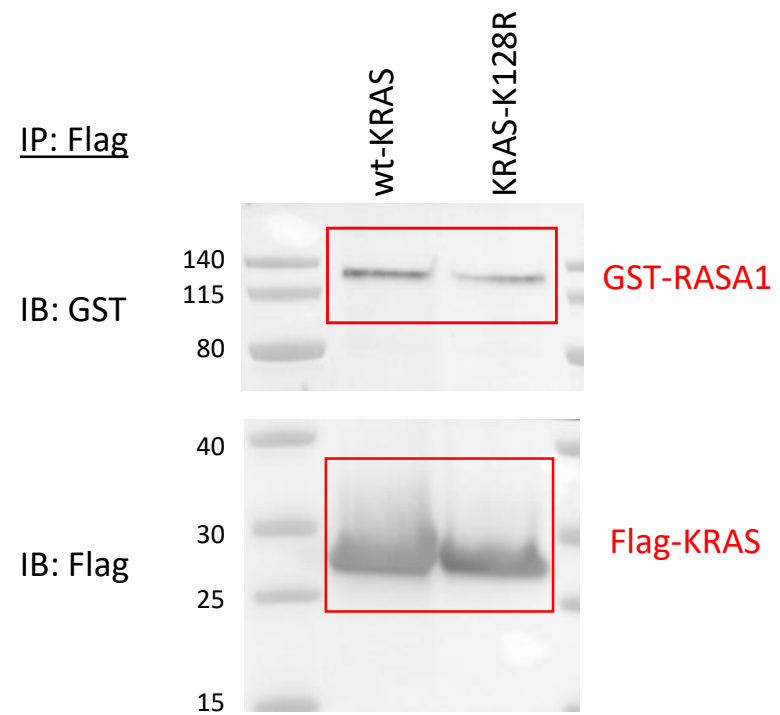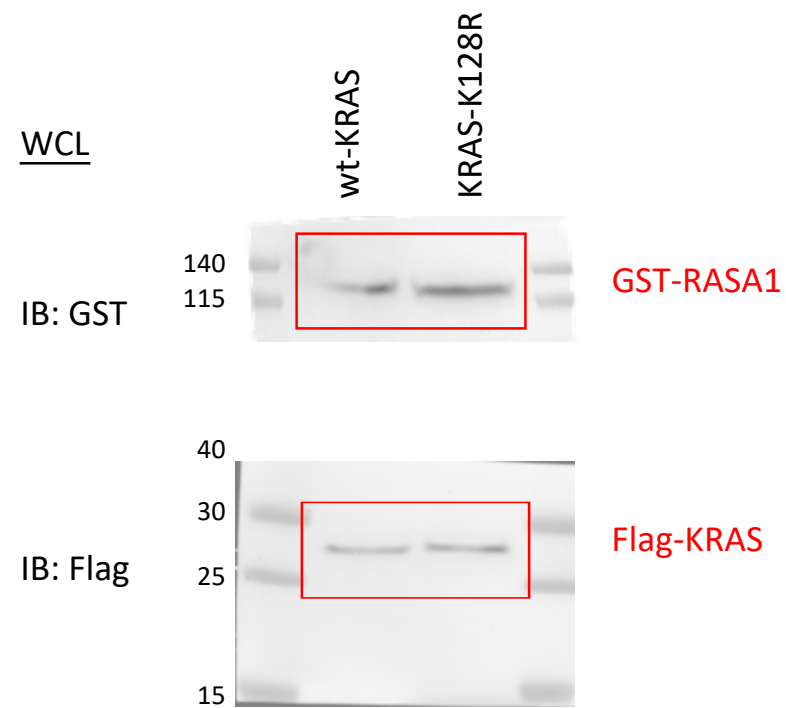

Supplement: Supplementary file 4 — Source data Fig. 2 [file 44318_2024_146_MOESM4_ESM.zip › Fig 2H-RASA1-KRAS-IP-WCL.pdf]

IP:Flag

WCL

140  
115  
80  
70  
50  
40

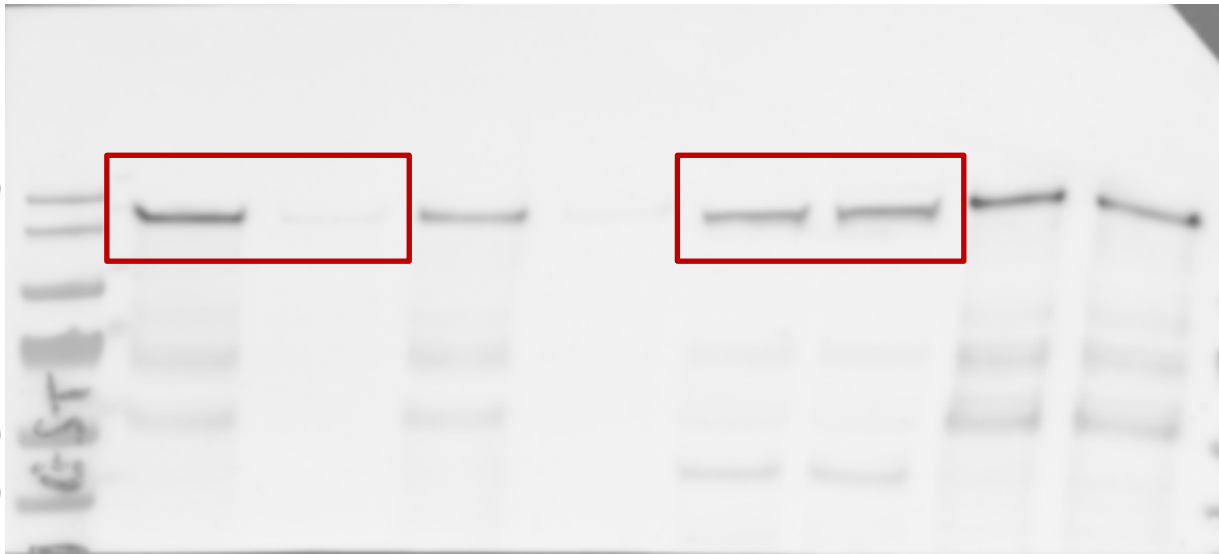

RASA1-GAP

30  
25  
15  
10

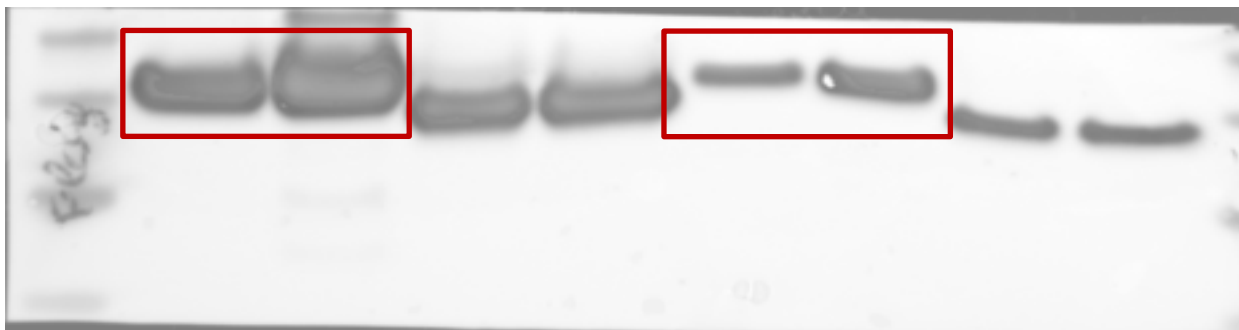

Flag-NRAS

Supplement: Supplementary file 4 — Source data Fig. 2 [file 44318_2024_146_MOESM4_ESM.zip › Fig 2H-RASA1-NRAS-IP-WCL.pdf]

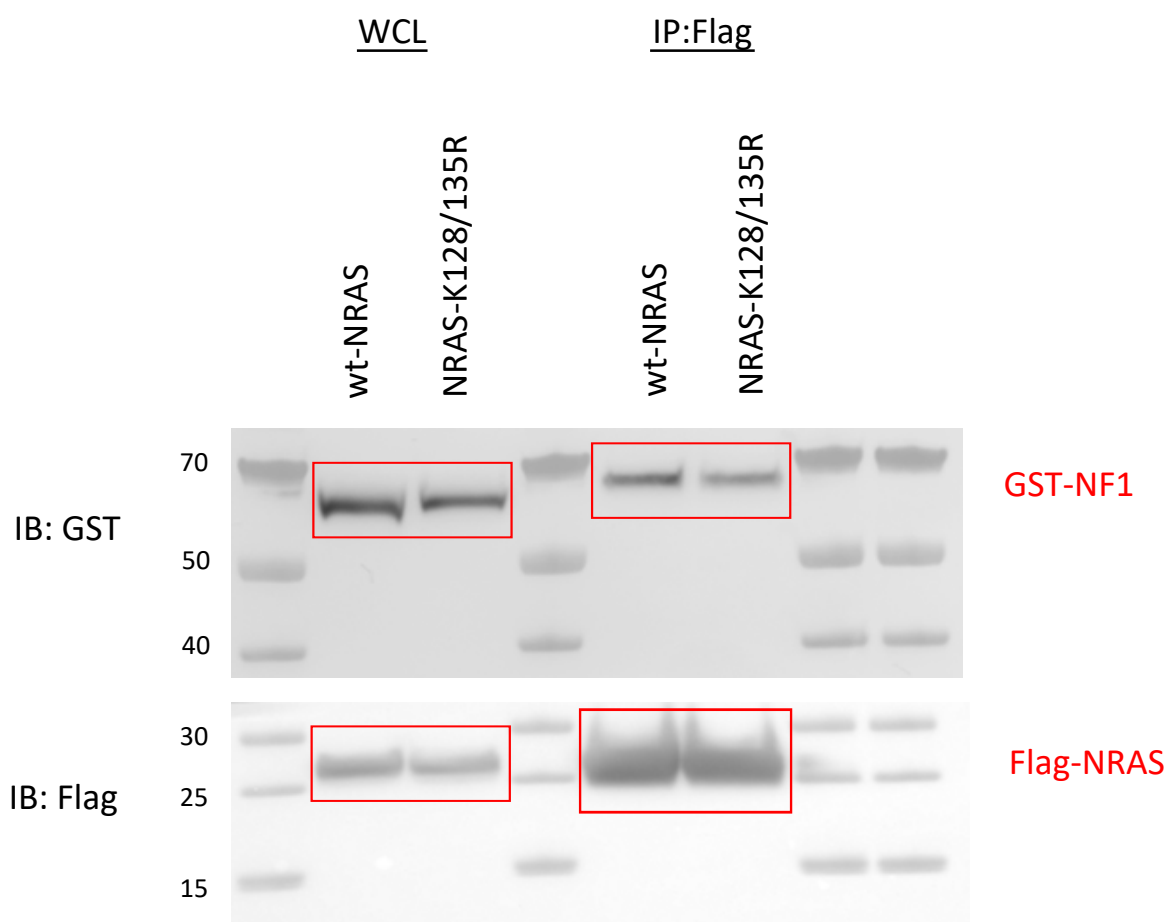

Supplement: Supplementary file 4 — Source data Fig. 2 [file 44318_2024_146_MOESM4_ESM.zip › Fig 2K-NF1-NRAS-IP-WCL.pdf]

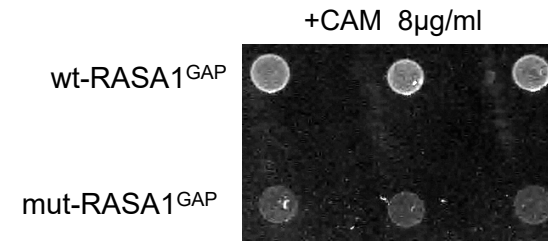

Supplement: Supplementary file 5 — Source data Fig. 3 [file 44318_2024_146_MOESM5_ESM.zip › Fig 3B-Growth assay.pdf]

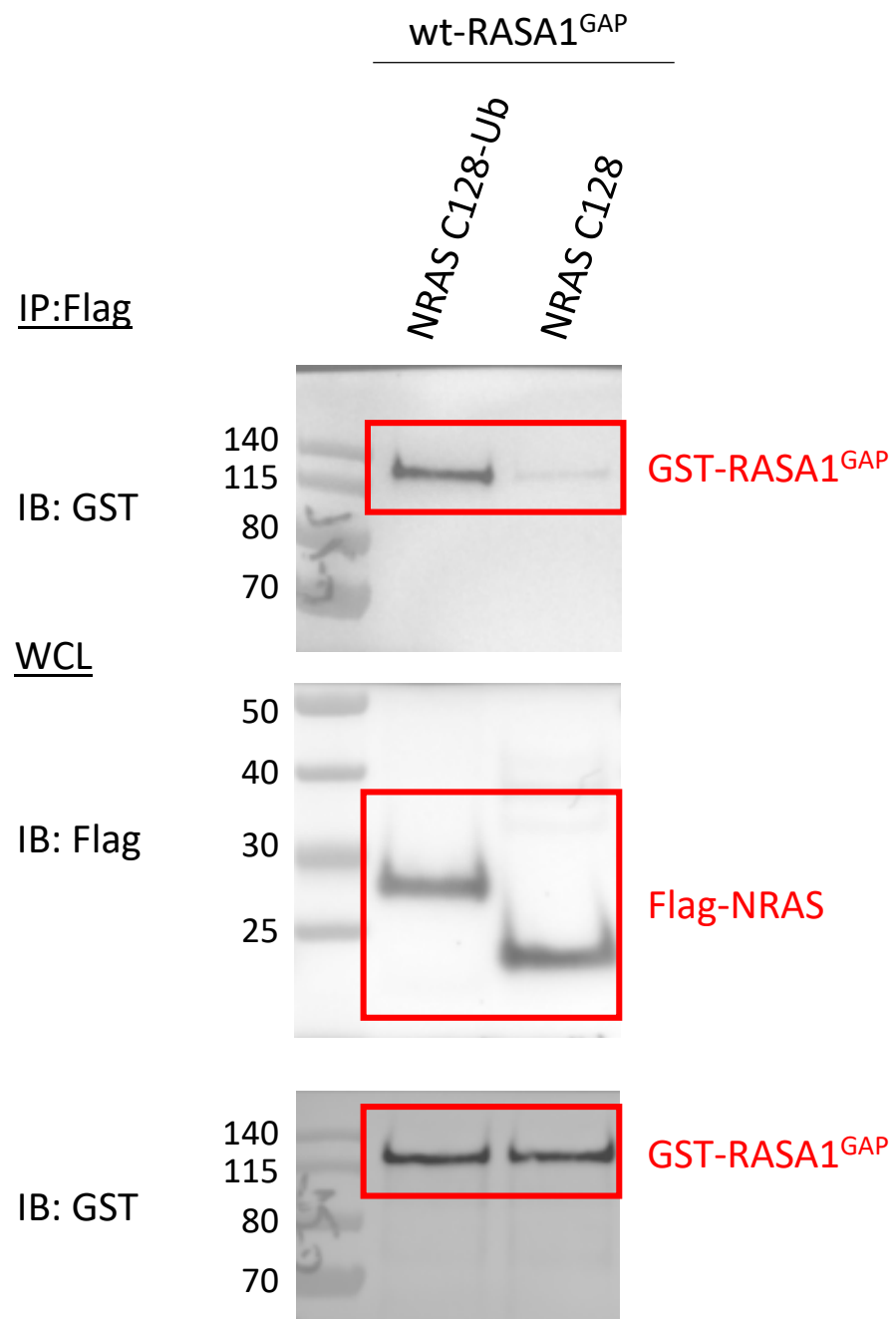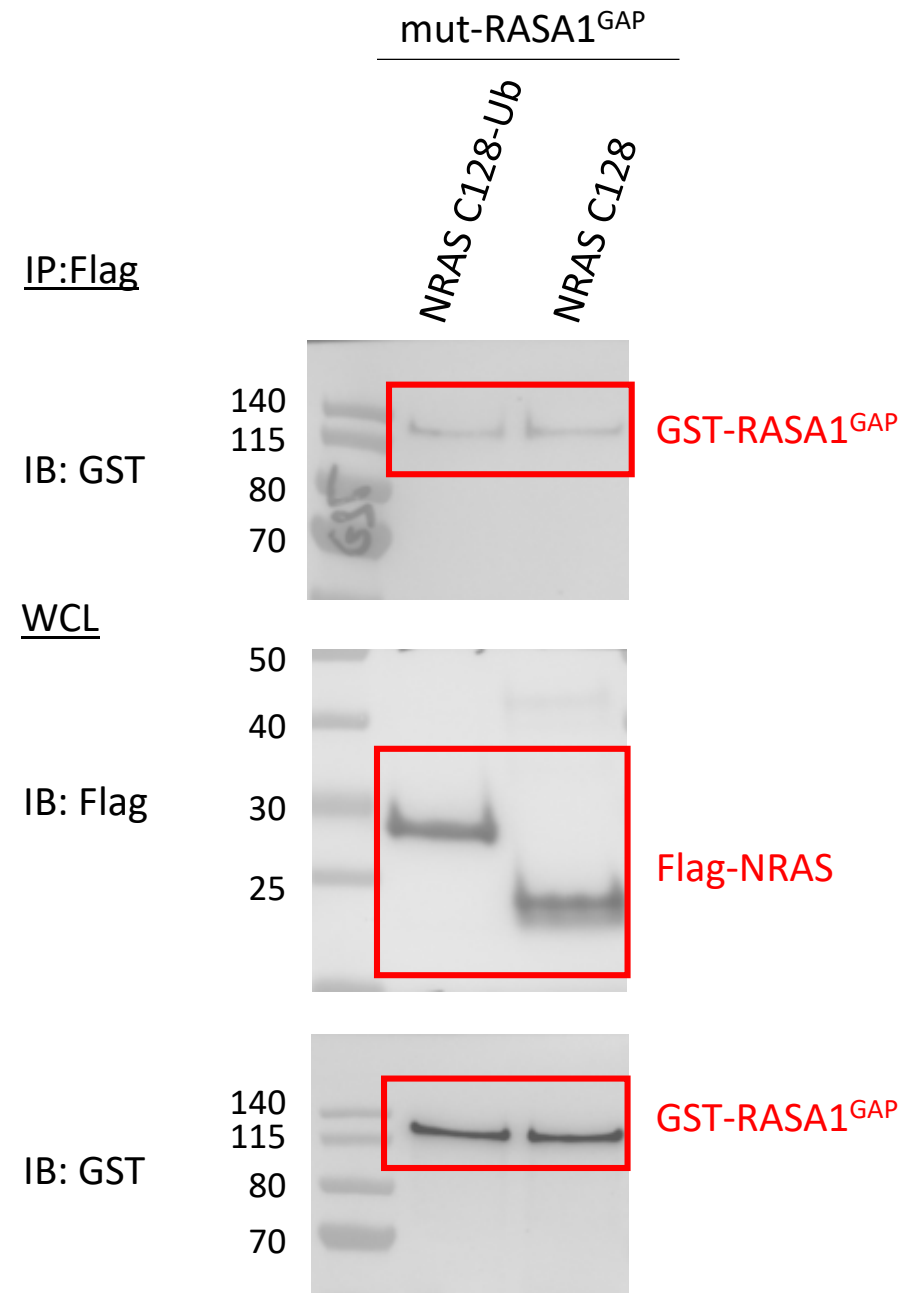

Supplement: Supplementary file 5 — Source data Fig. 3 [file 44318_2024_146_MOESM5_ESM.zip › Fig 3C-western IP-WCL.pdf]

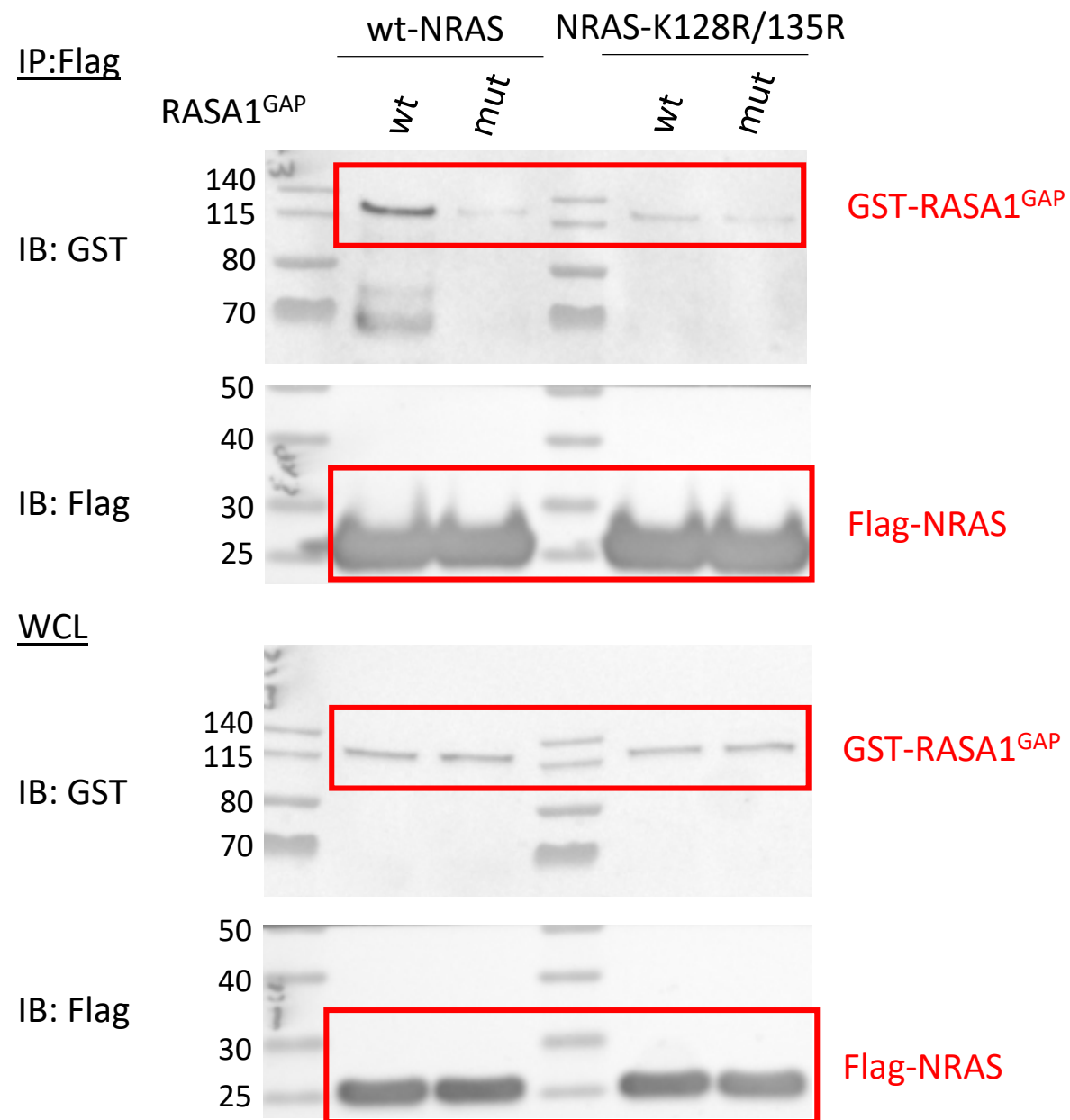

Supplement: Supplementary file 5 — Source data Fig. 3 [file 44318_2024_146_MOESM5_ESM.zip › Fig 3D-western IP-WCL.pdf]

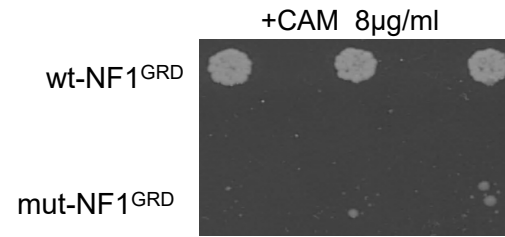

Supplement: Supplementary file 5 — Source data Fig. 3 [file 44318_2024_146_MOESM5_ESM.zip › Fig 3F-Growth assay.pdf]

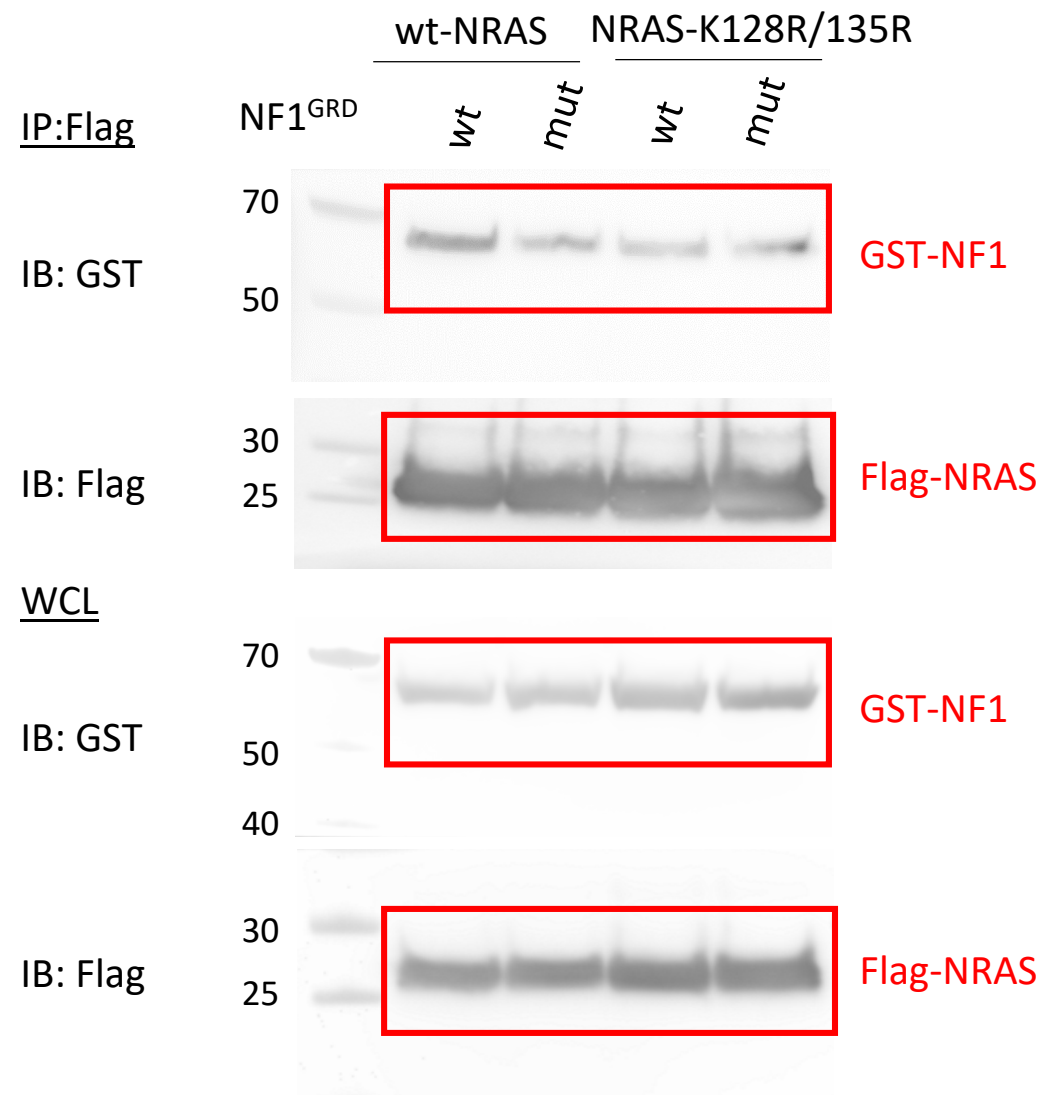

Supplement: Supplementary file 5 — Source data Fig. 3 [file 44318_2024_146_MOESM5_ESM.zip › Fig 3G-western IP-WCL.pdf]

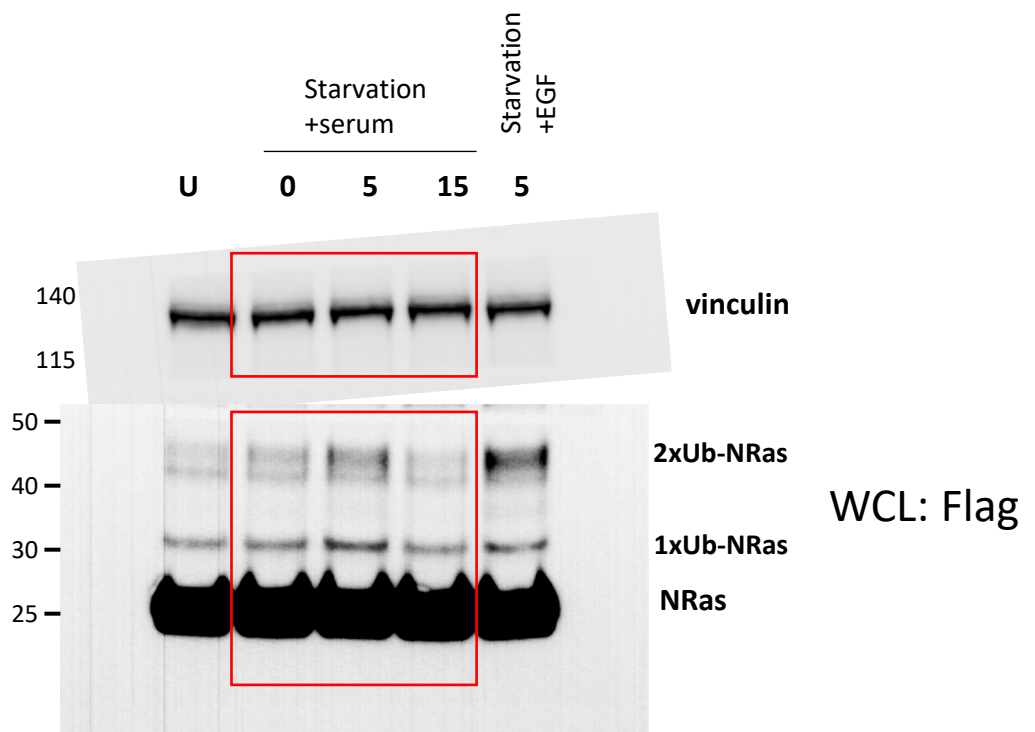

Supplement: Supplementary file 6 — Source data Fig. 4 [file 44318_2024_146_MOESM6_ESM.zip › Fig 4B-Ub-NRAS-serum-WCL.pdf]

| Serum (min) | wt-NRAS |   |    | NRAS K128/135R |   |    |
|-------------|---------|---|----|----------------|---|----|
|             | 0       | 5 | 20 | 0              | 5 | 20 |

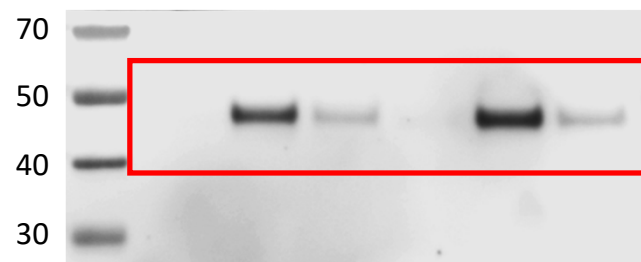

p-MEK1/2 (S217/221)

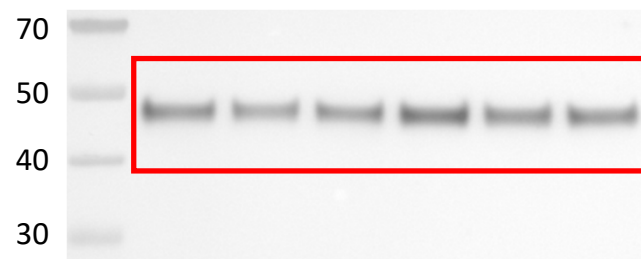

MEK1/2

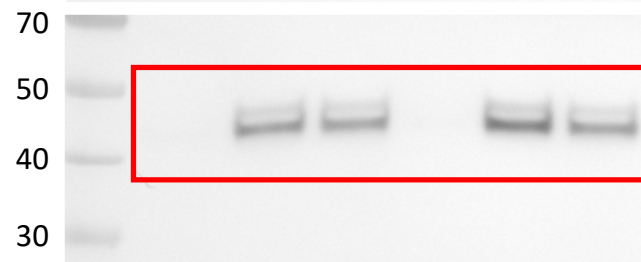

p-ERK1/2 (T202/Y204)

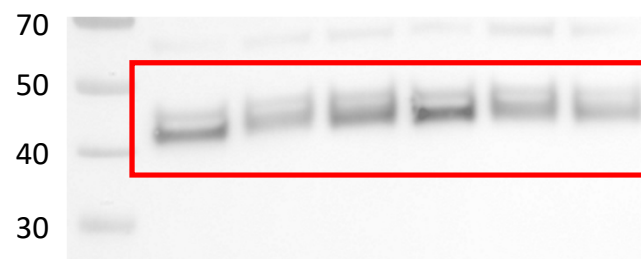

ERK1/2

| Serum (min) | wt-KRAS |   |    | KRAS K128R |   |    |
|-------------|---------|---|----|------------|---|----|
|             | 0       | 5 | 20 | 0          | 5 | 20 |

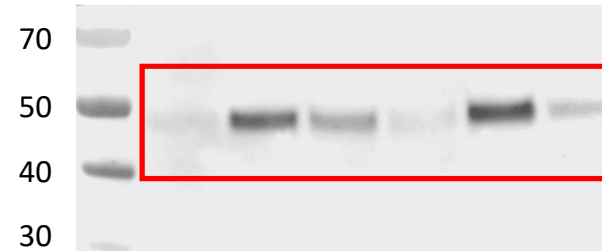

p-MEK1/2 (S217/221)

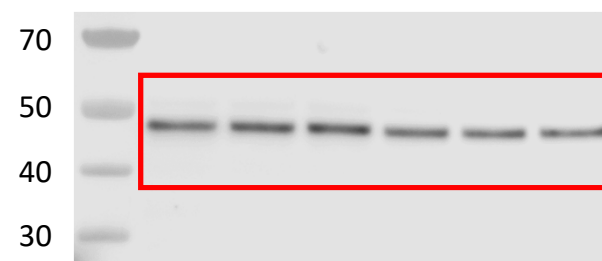

MEK1/2

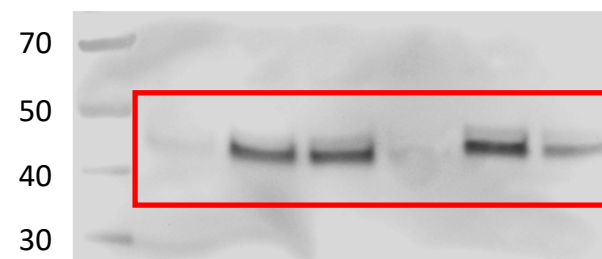

p-ERK1/2 (T202/Y204)

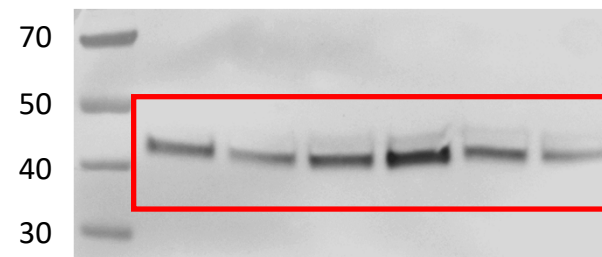

ERK1/2

Supplement: Supplementary file 6 — Source data Fig. 4 [file 44318_2024_146_MOESM6_ESM.zip › Fig 4E-western.pdf]

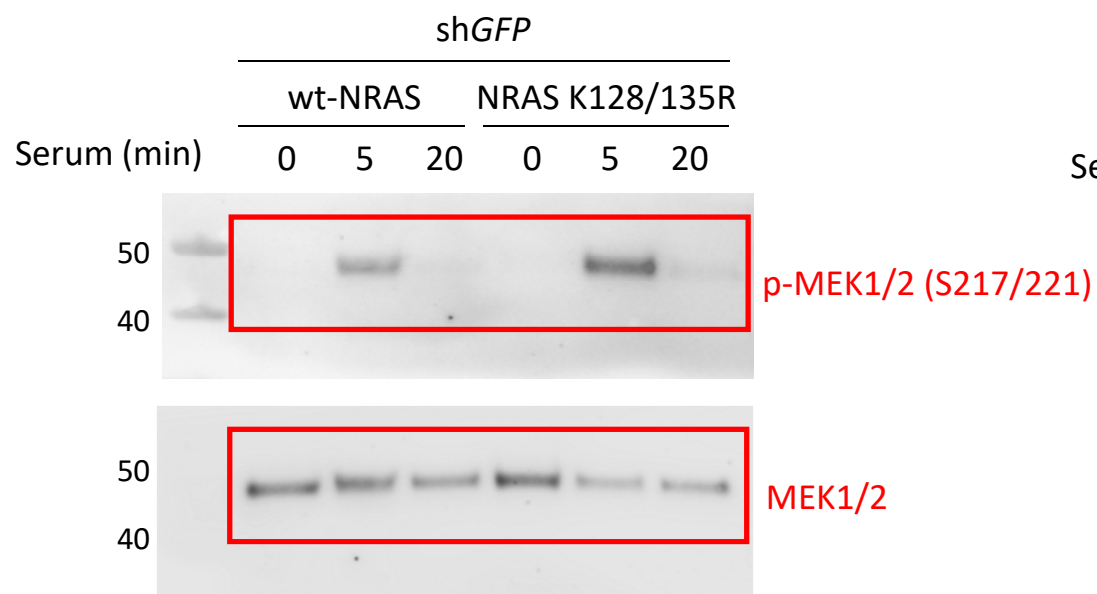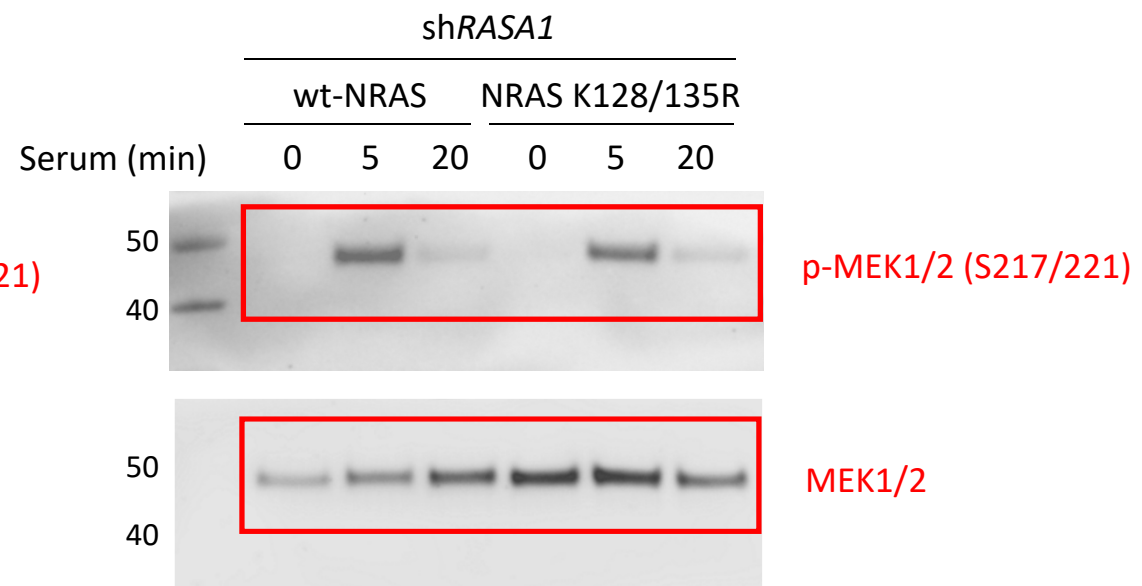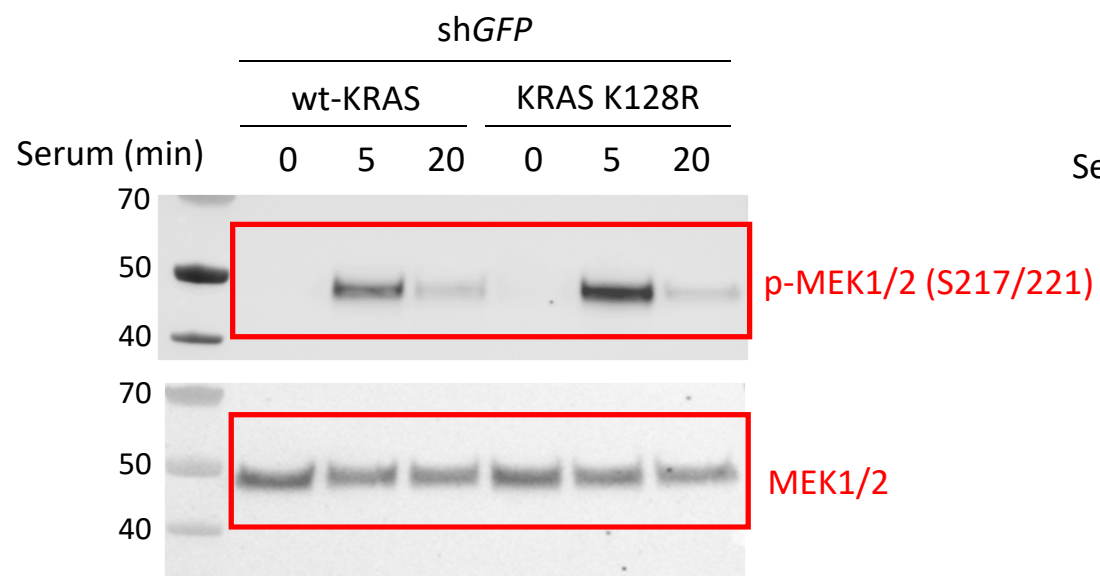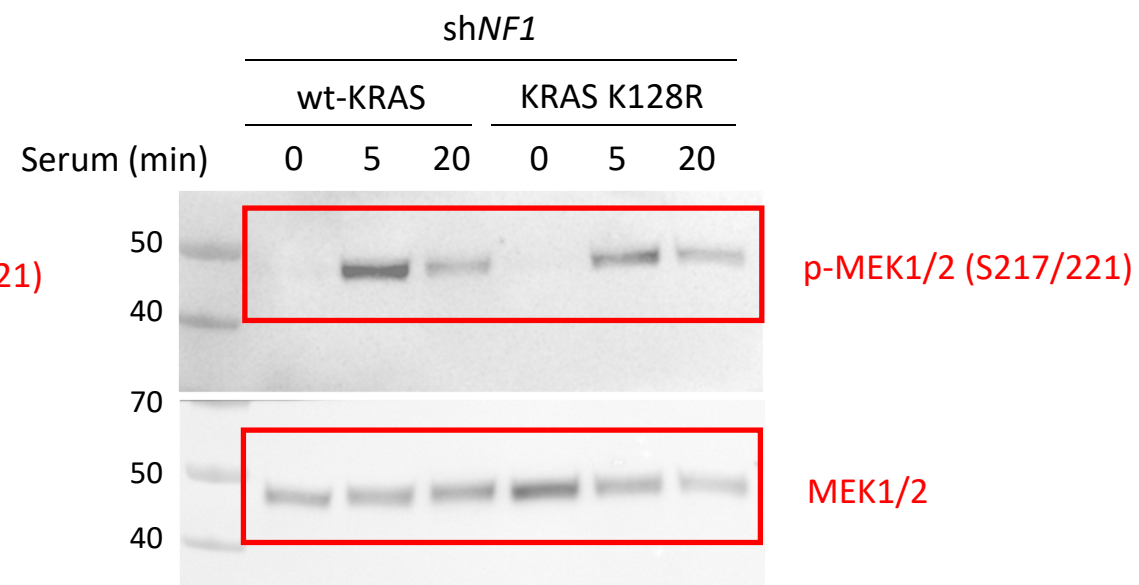

Supplement: Supplementary file 6 — Source data Fig. 4 [file 44318_2024_146_MOESM6_ESM.zip › Fig 4F-western.pdf]

### KRAS-G12D

### KRAS-G12D/K128R

Rep 1

Rep 2

Rep 1

Rep 2

**Clone 3**

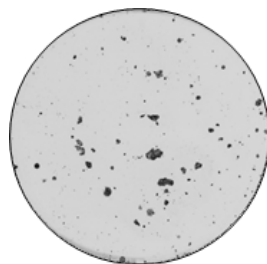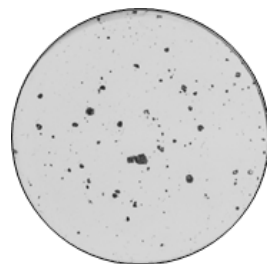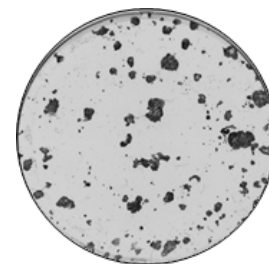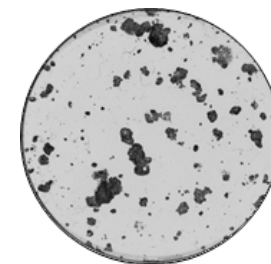

**Clone 4**

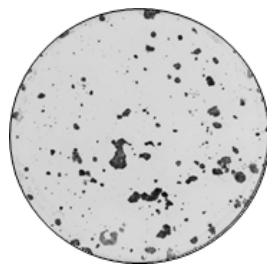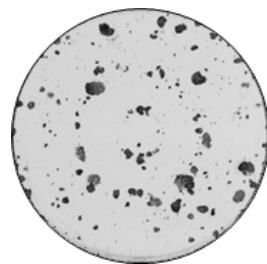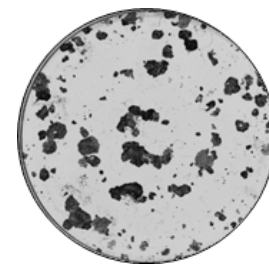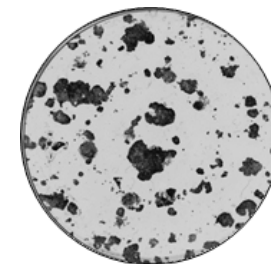

**Clone 5**

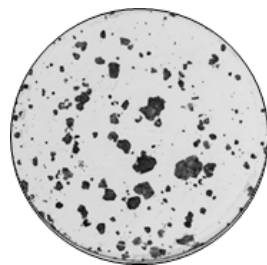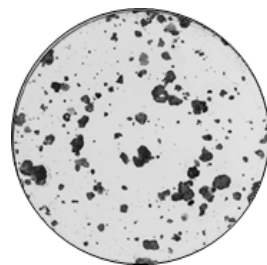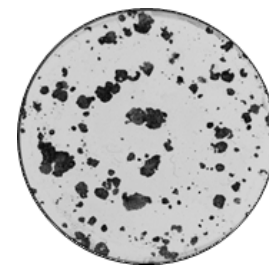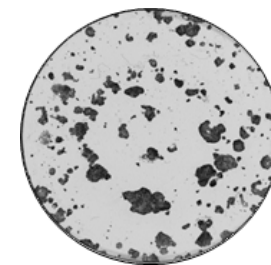

Supplement: Supplementary file 7 — Source data Fig. 5 [file 44318_2024_146_MOESM7_ESM.zip › Fig 5B-2D colony.pdf]

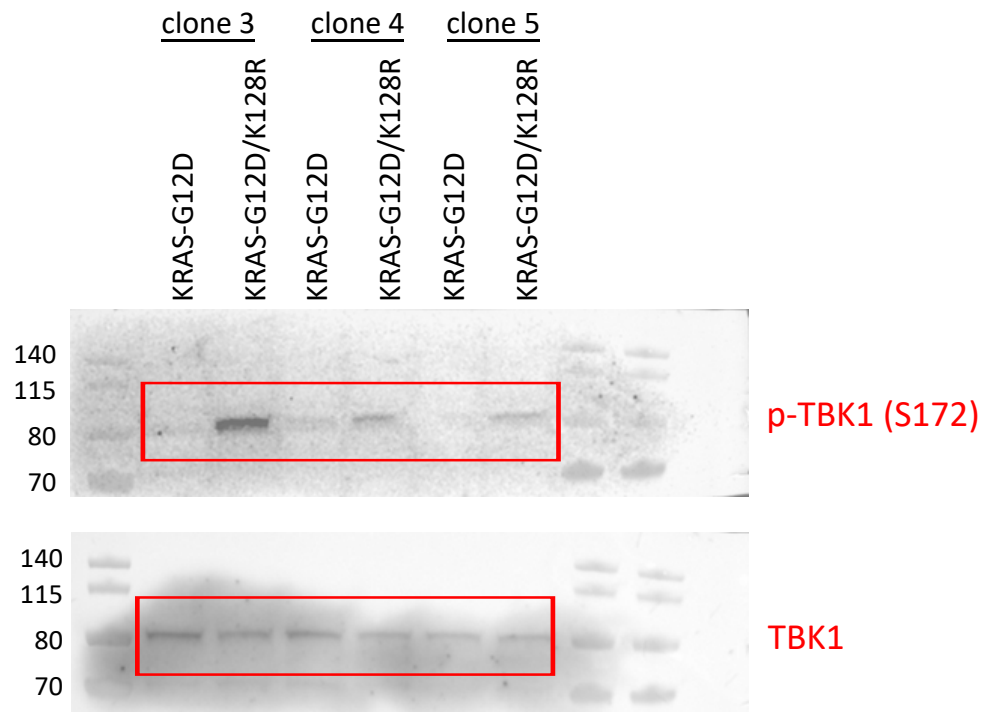

Supplement: Supplementary file 7 — Source data Fig. 5 [file 44318_2024_146_MOESM7_ESM.zip › Fig 5E-western.pdf]

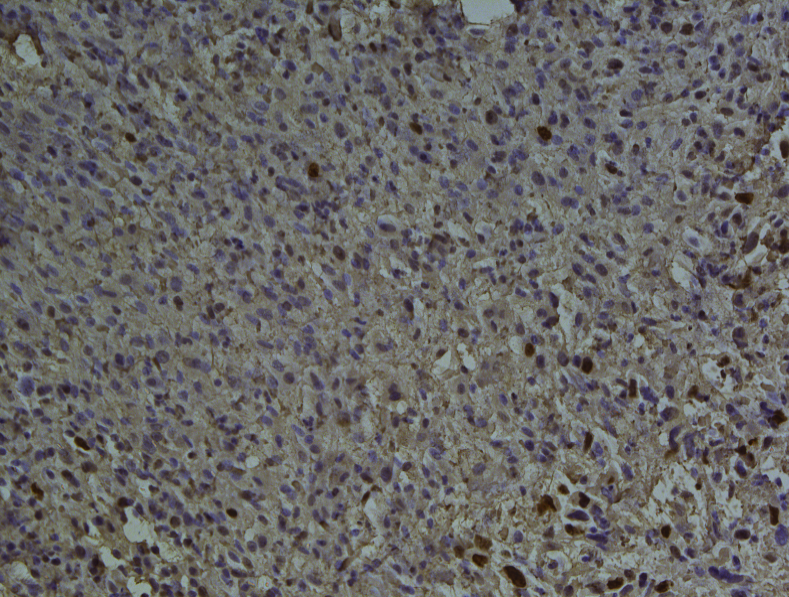

Supplement: Supplementary file 7 — Source data Fig. 5 [file 44318_2024_146_MOESM7_ESM.zip › Fig 5F-pTBK1-G12D.tif]

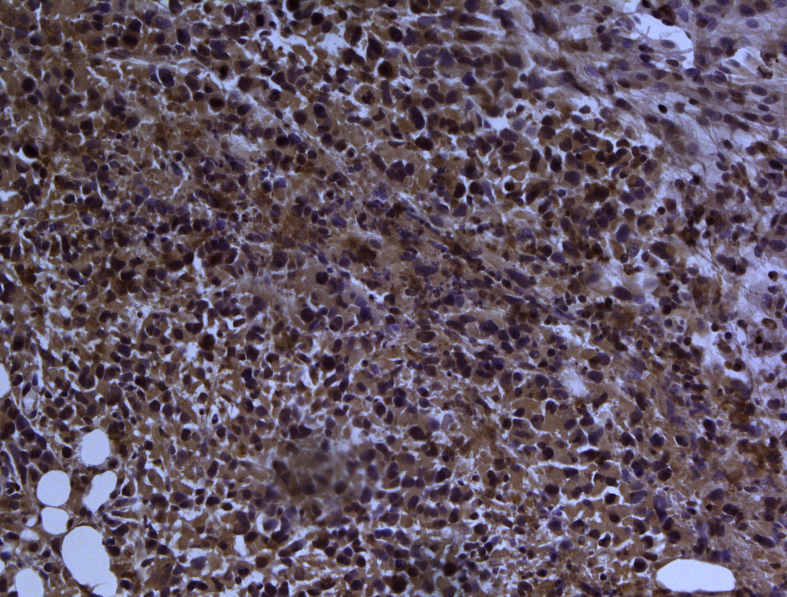

Supplement: Supplementary file 7 — Source data Fig. 5 [file 44318_2024_146_MOESM7_ESM.zip › Fig 5F-pTBK1-G12D-K128R.tif]

IP:Sec5

KRAS-G12D  
KRAS-G12D/K128R

IB: Sec5

140  
115  
80  
70

SEC5

IB: RalB

30  
25  
15

RalB-GTP

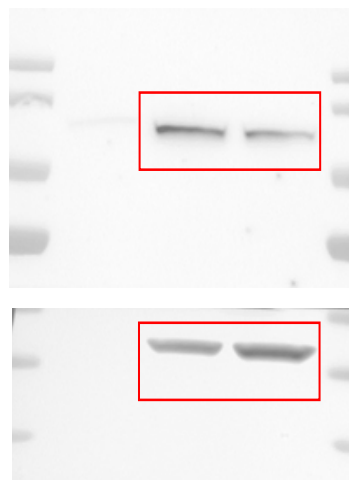

Supplement: Supplementary file 7 — Source data Fig. 5 [file 44318_2024_146_MOESM7_ESM.zip › Fig 5G-western IP.pdf]

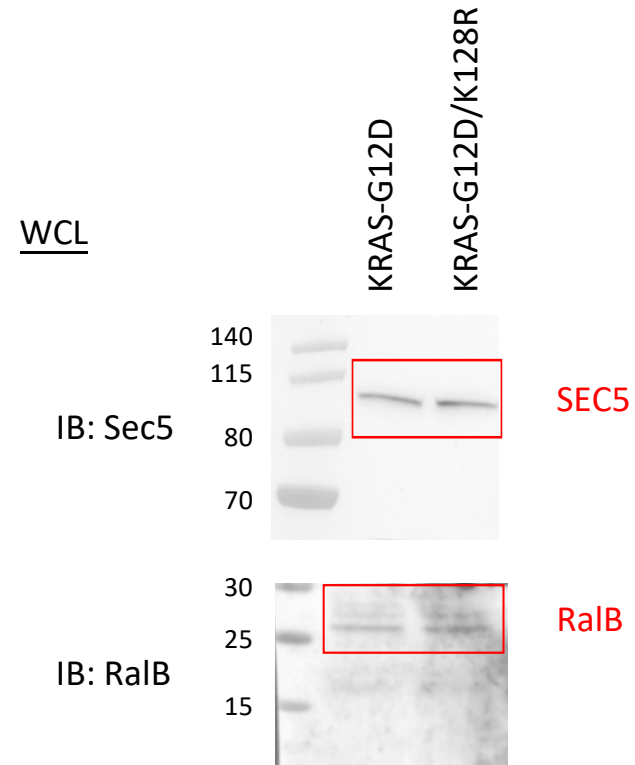

Supplement: Supplementary file 7 — Source data Fig. 5 [file 44318_2024_146_MOESM7_ESM.zip › Fig 5G-western WCL.pdf]

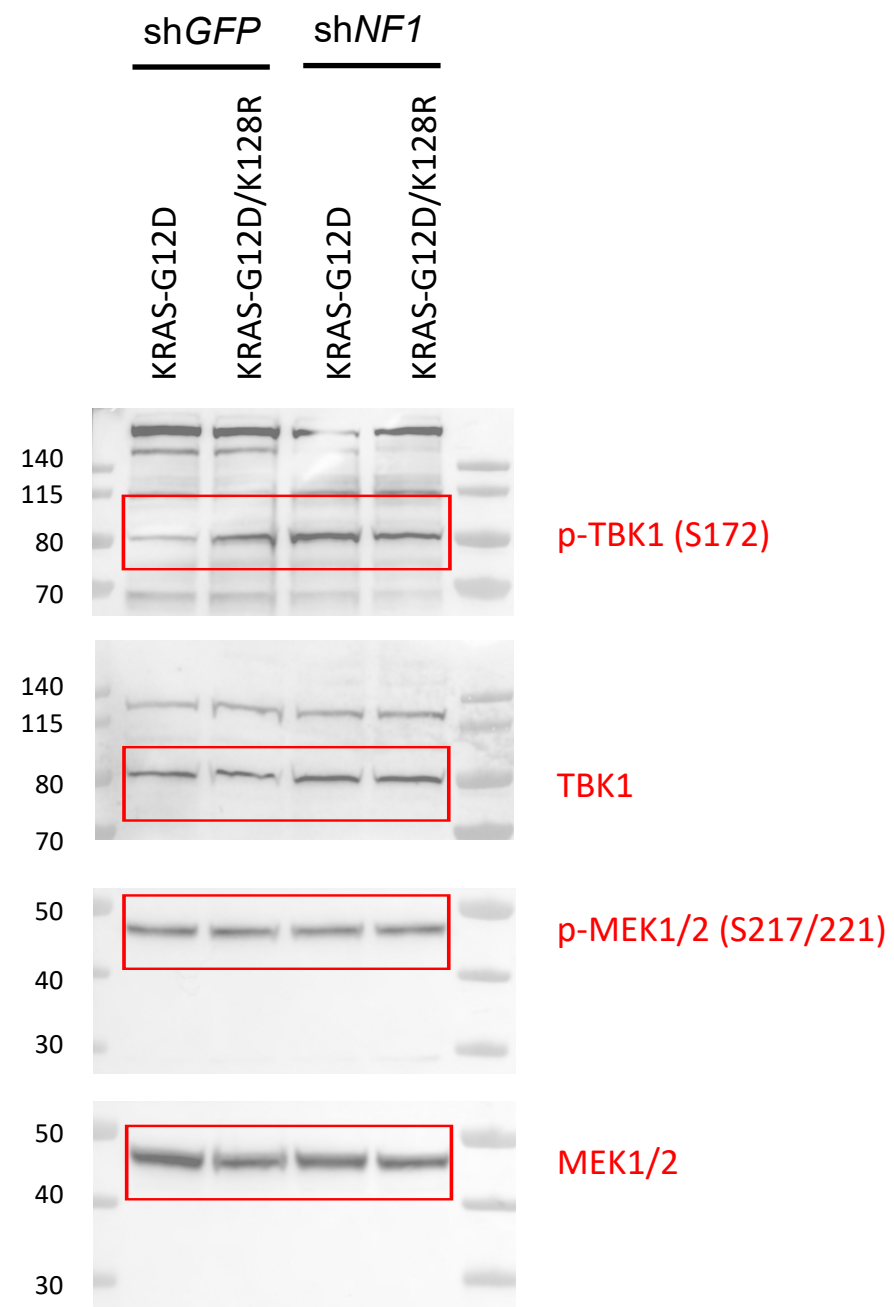

Supplement: Supplementary file 7 — Source data Fig. 5 [file 44318_2024_146_MOESM7_ESM.zip › Fig 5H-western.pdf]
