# Supplementary material for: K128 ubiquitination constrains RAS activity by expanding its binding interface with GAP proteins
Source: EMBO J. 2024 Jun 10;43(14):2862–77. doi: 10.1038/s44318-024-00146-w (PMC11251195; doi:10.1038/s44318-024-00146-w)
Supplement: Supplementary file 4 — Source data Fig. 2 [file 44318_2024_146_MOESM4_ESM.zip › Fig 2K-NF1-KRAS-IP-WCL.pdf]

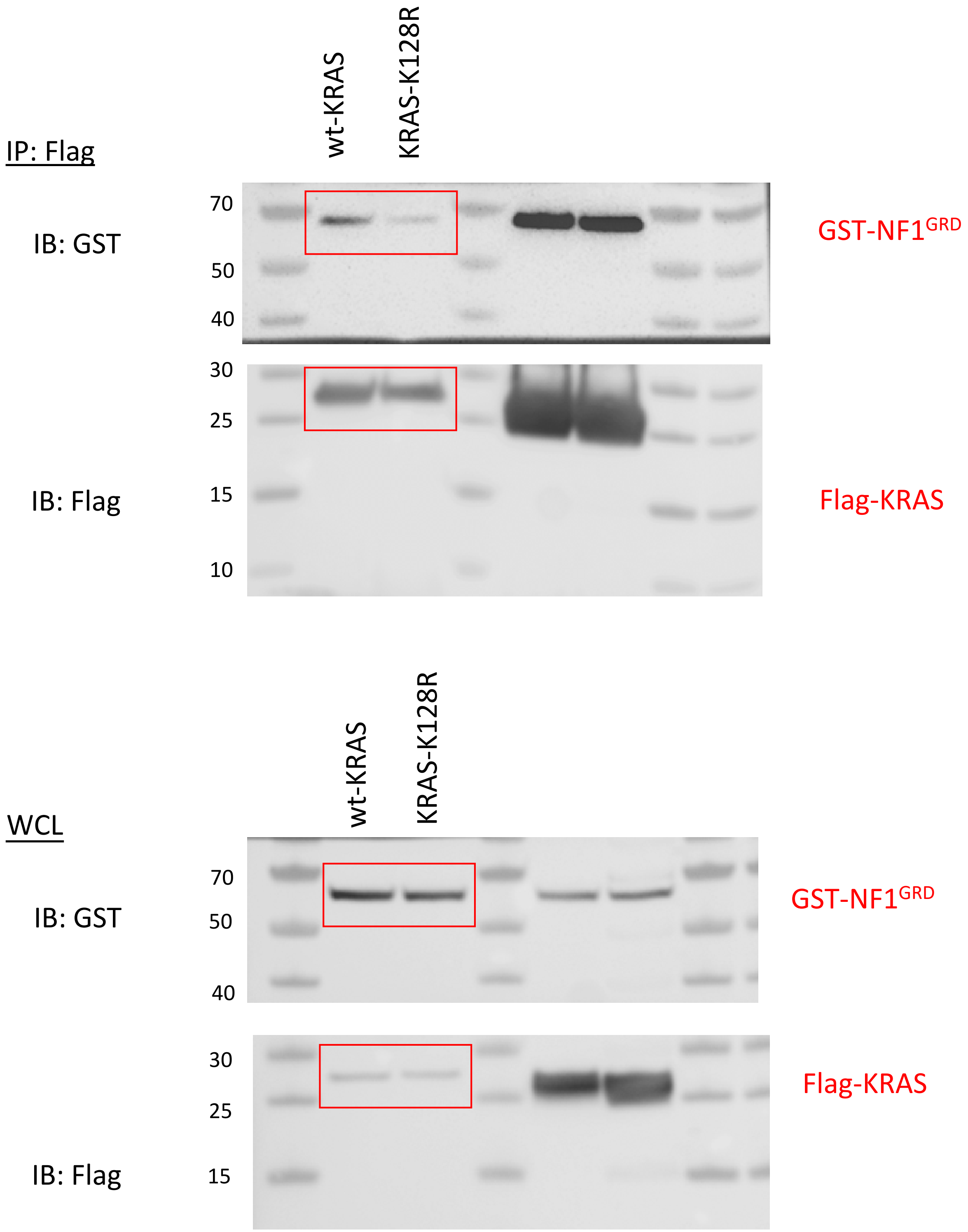

IP: Flag
wt-KRAS
KRAS-K128R
IB: GST
70
50
40
GST-NF1GRD
30
25
15
10
IB: Flag
Flag-KRAS
WCL
wt-KRAS
KRAS-K128R
IB: GST
70
50
40
GST-NF1GRD
30
25
15
IB: Flag
Flag-KRAS
